# Upcycling of Decommissioned Wind Turbine Blades: An Investigation of Stress Distributions in Glass Fiber-Reinforced Polymers Beams

**DOI:** 10.3390/ma19173622

**Published:** 2026-08-26

**Authors:** Changlang Wu, Jacob Wittrup Schmidt, Dario Parigi

**Affiliations:** 1 Department of Built Environment, Aalborg University, 9220 Aalborg, Denmark; 2 Civil Engineering and the Green Transition in the Built Environment (CEBE) RF3 Low-Carbon and Construction Materials, Department of Built Environment, Aalborg University, 9220 Aalborg, Denmark

**Keywords:** decommissioned wind turbine blades, GFRP composite, GL-FRP beam, upcycling, stress distribution

## Abstract

Wind turbines are among the most widely adopted renewable energy systems, yet the end-of-life management of wind turbine blades remains a major challenge. The turbine blades are primarily made of glass fiber-reinforced polymers (GFRP), which are difficult to recycle without degrading their inherent structural integrity. Instead of shredding or downcycling, this study explores a reuse strategy that preserves intact laminate from decommissioned blades and repurposes them into structural beams via glue-laminated fiber-reinforced polymers (GL-FRP) assemblies. This concept was implemented using commercial GFRP for blade-derived laminates, focusing on the structural feasibility rather than material sourcing. Adhesive joints were designed as the key enabling mechanism for structural reuse and characterized under tensile loading. Building on this, the flexural performance of GL-FRP beams was investigated in horizontal and vertical configurations, yielding an ultimate load-bearing capacity of 8 kN and 20 kN, respectively. Furthermore, stress distributions obtained from experiments, finite element (FE) simulations, and analytical frameworks were evaluated and compared. The results highlight the governing role of interlaminar stress in determining structural performance and failure of the horizontal beam. The findings provide a foundational understanding of repurposing decommissioned wind turbine blades as structural elements, offering a novel strategy for material upcycling in wind energy sector and contributing to the development of circular construction solutions.

## 1. Introduction

**Figure 1:**
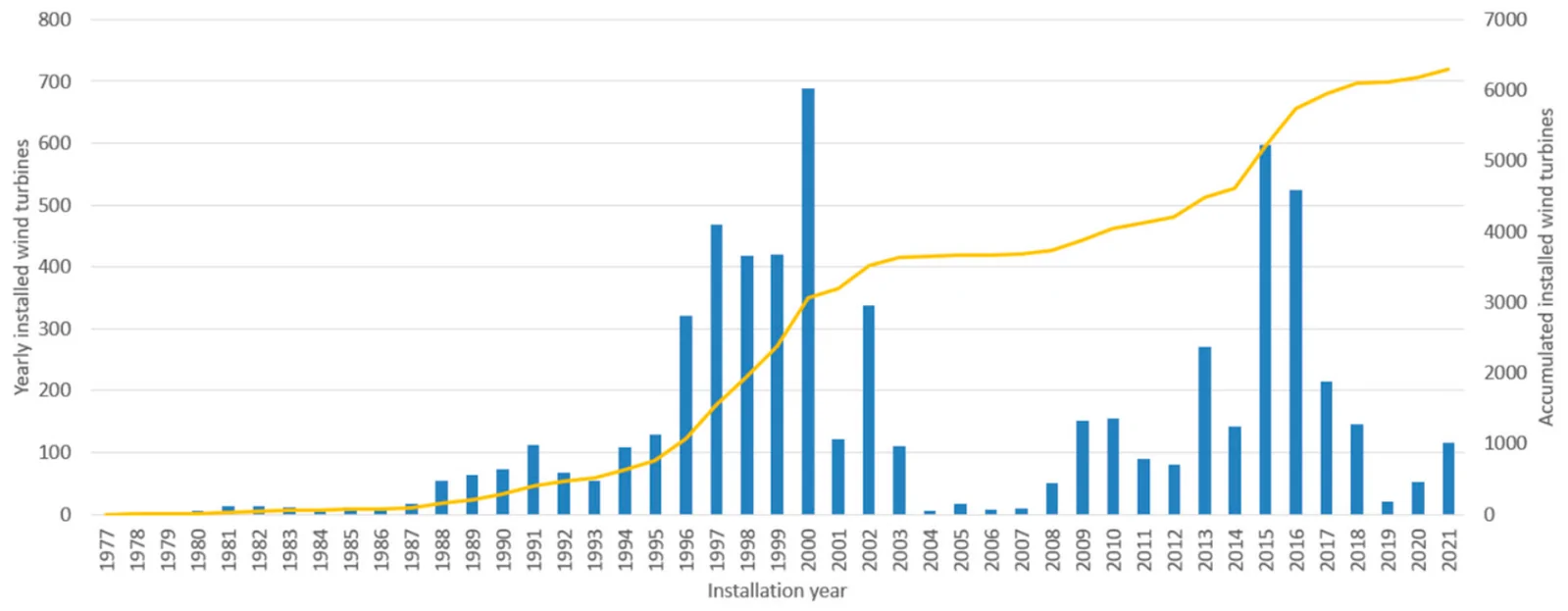
Existing wind turbines in Denmark and their installation year [4].

Wind energy has experienced rapid development over the past few decades and has emerged as one of the most promising, cost-effective, and environmentally friendly sources of renewable energy [1]. Wind turbines, which play a pivotal role in this energy transformation, are designed with an operational lifespan of 20 to 25 years [2]. Taking Denmark as an example, there was approximately 6300 wind turbines existing in the country at the end of 2021 (see Figure 1). A large percentage of these turbines are from 20 to 25 years old, approaching the end of their operational lifetime. Nevertheless, they only account for a small fraction of the total wind turbines installed across Europe [3]. As an increasing number of wind turbines reach the end of their operational lifespan, sustainable strategies for their end-of-life management have become a critical focus in the wind energy sector.

**Figure 2:**
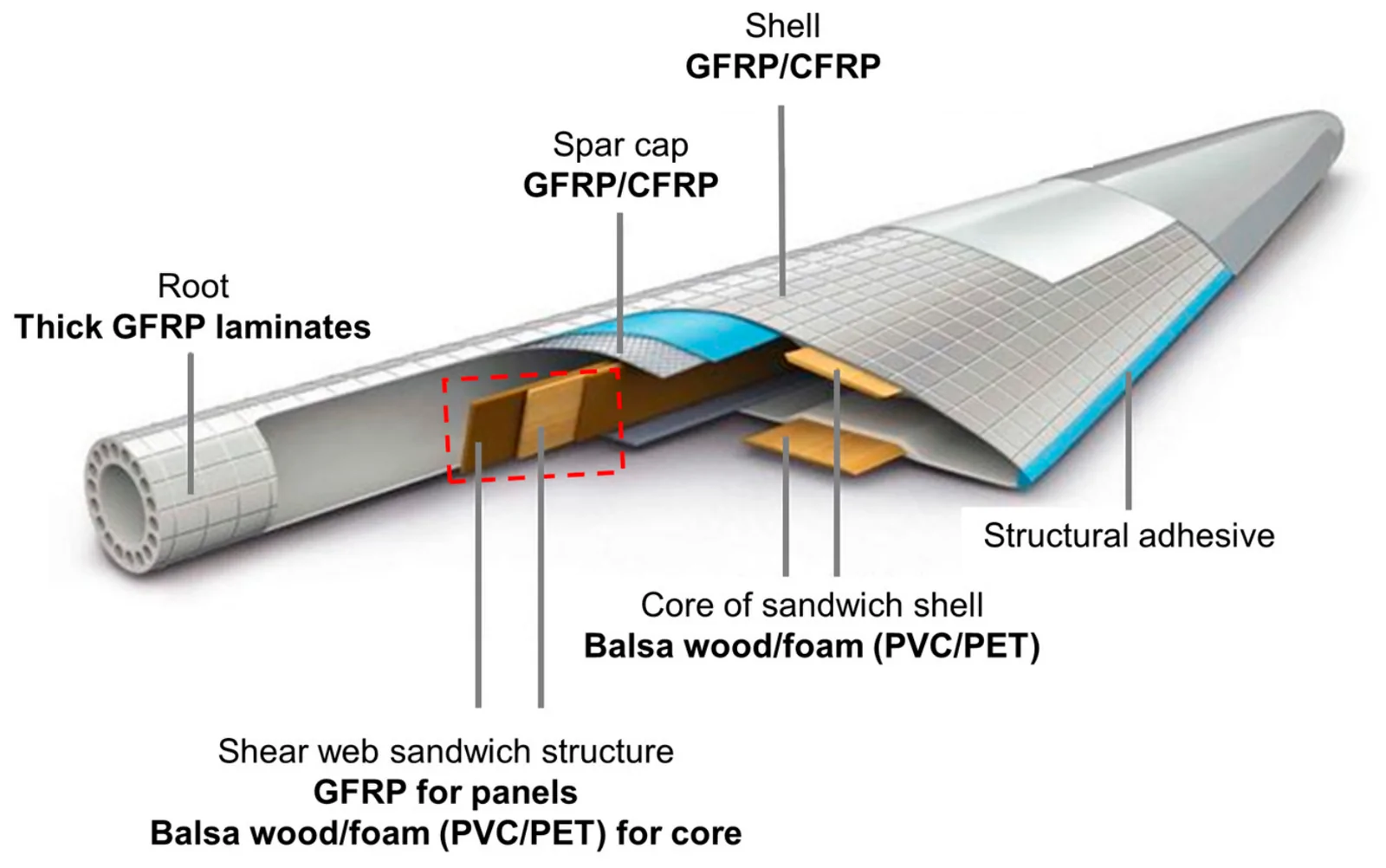
An anatomy of a modern wind turbine blade, showing the main components and the corresponding materials [11].

While wind turbines are among the most widely utilized renewable energy technologies, end-of-life management remains a significant challenge [5]. This is primarily associated with the wind turbine blades [6]. Figure 2 illustrates an internal structural configuration of a modern turbine blade. Wind turbine blades are manufactured predominantly from glass fiber-reinforced polymer (GFRP), while other materials are incorporated to meet specific design requirements. For instance, core sections of the shear-web sandwich structures commonly employ end-grain balsa wood or polyvinyl-chloride (PVC) foam because of their high stiffness-to-weight ratio [7,8]. In regions subjected to critical load, such as the spar cap, carbon fiber-reinforced polymer (CFRP) is substituted for GFRP to provide higher stiffness and strength at a lower weight [9]. Owing to its advantageous combination of mechanical performance, long-term durability, manufacturing efficiency, and cost, GFRP remains the principal material used in modern wind-turbine blade construction [5]. However, GFRP is typically based on thermoset resins. Their cross-linked polymer networks cannot be remelted or reshaped [10], making conventional recycling methods like melting and remolding impractical.

Current recycling methods are mainly categorized into thermal, chemical, and mechanical processes [12]. Table 1 summarizes the advantages and limitations of different recycling technologies. Thermal methods, including pyrolysis and fluidized-bed processing, use high-temperature decomposition to recover fibers; however, the recovered fibers often exhibit reduced mechanical properties due to thermal degradation [13]. Chemical recycling methods—including solvolysis, hydrothermal liquefaction, supercritical fluid treatment, and solvent dissolution—have gained increasing attention due to their ability to recover high-quality fibers and convert resin matrix into reusable chemical products [14]. Nevertheless, these methods are often limited by high energy consumption [15], complex product separation, and limited economic feasibility on a commercial scale [16].

Mechanical recycling refers to the physical size reduction of fiber-reinforced composites without altering their chemical composition [17]. So far, it has been considered the most industrially mature and cost-effective recycling approach. Grinding and shredding are a type of mechanical recycling method that produces finely ground, fibrous or particulate materials. These materials could be used as aggregate in concrete/asphalt [18], reinforcement in 3D printing [19], etc. Another solution for the decommissioned wind turbine blades is to repurpose or upcycle them in their original structural form instead of shredding. This approach avoids energy-intensive processing and utilizes the residual performance of the turbine blade to extend its lifespan [20]. As the volume of decommissioned wind turbine blades continues to increase, enhancing the efficiency and environmental sustainability of mechanical recycling has become an essential topic in both academia and industry.

**Table 1:** A comparison among current recycling technologies for decommissioned wind turbine blades.

Recycling Techniques		Advantages	Limitations
Thermal methods	Pyrolysis process	Resin decomposition without combustion, fuel-rich byproduct, large-scale operation [1,21]	Fiber damage and low quality, residual resin on the fiber, high energy consumption [21,22,23]
	Fluidized bed process	Cleaner surface of recycled fiber, large-scale operation [24]	Reduced fiber length and mechanical properties of recycled fiber, limited recovering of resin, emission of pollutants [24,25]
Chemical methods	Supercritical fluid method	High-quality fiber recovery [26], selective degradation [16]	Lab-scale, costly, limited efficiency in fiber recovery, extreme operational condition [27]
	Solvent dissolution method	Effective matrix removal, relatively low temperature process [28]	Lab-scale, long process time, use of hazardous chemicals [29]
	Hydrothermal liquefaction method	Effective removal of matrix, high-quality of fiber recovery, no harsh chemicals/toxic emission [14]	Lab-scale, high energy consumption, complex byproducts require further refining/separation [16]
Mechanical methods	Grinding	Easy to transport and handle, relatively simple, can be used to replace virgin raw materials [30]	Downcycling, dust and fiber issues [31], high energy consumption [32]
	Shredding	Easy to transport and handle, essential step before grinding [33]	Noise, equipment wear, high energy consumption [34]
	Repurposing section/whole blade	Preservation of fiber integrity [13,35], minimize processing cost	Difficulty in transportation/handling, limited applications, design constraints [16]

In the last decade, large sections of decommissioned wind turbine blades have been repurposed for architectural or other structural applications. One of the most explored solutions is to reuse the turbine blade for primary load-bearing girders in bridges. Over the past decade, academic and industrial research in this domain has progressed from early conceptual feasibility frameworks [36] and numerical modeling to full-scale empirical prototyping [37] and successful real-world field deployments [38]. In addition to bridge girders, other applications in heavy civil infrastructure include noise barriers [39], transmission poles [40], trench protection wall [41], and wave energy converter [42] etc. Various design-led projects and industrial pilots have also demonstrated using turbine blades for bicycle shelters [43], playgrounds [44], and public benches [45]. However, the unique geometry of turbine blades and the logistical challenges of handling and transport limit their large-scale and standardized production [20]. To overcome these challenges, researchers and industry partners have been exploring the reuse of cut-out blade sections. Case studies have demonstrated the feasibility of reusing the cut-out sections to build picnic tables [46] and indoor furniture [47]. Building on this concept, some studies highlighted the potential of repurposing cut-out sections as structural elements considering their high strength and stiffness [48]. While these initiatives divert the turbine blade waste from landfills, they predominantly reuse the material for low-value, customized, low-scalability secondary products. To date, no systematic research has established a high-value recycling framework that is capable of upcycling wind turbine blades for standardized, load-bearing elements for building construction.

**Figure 3:**
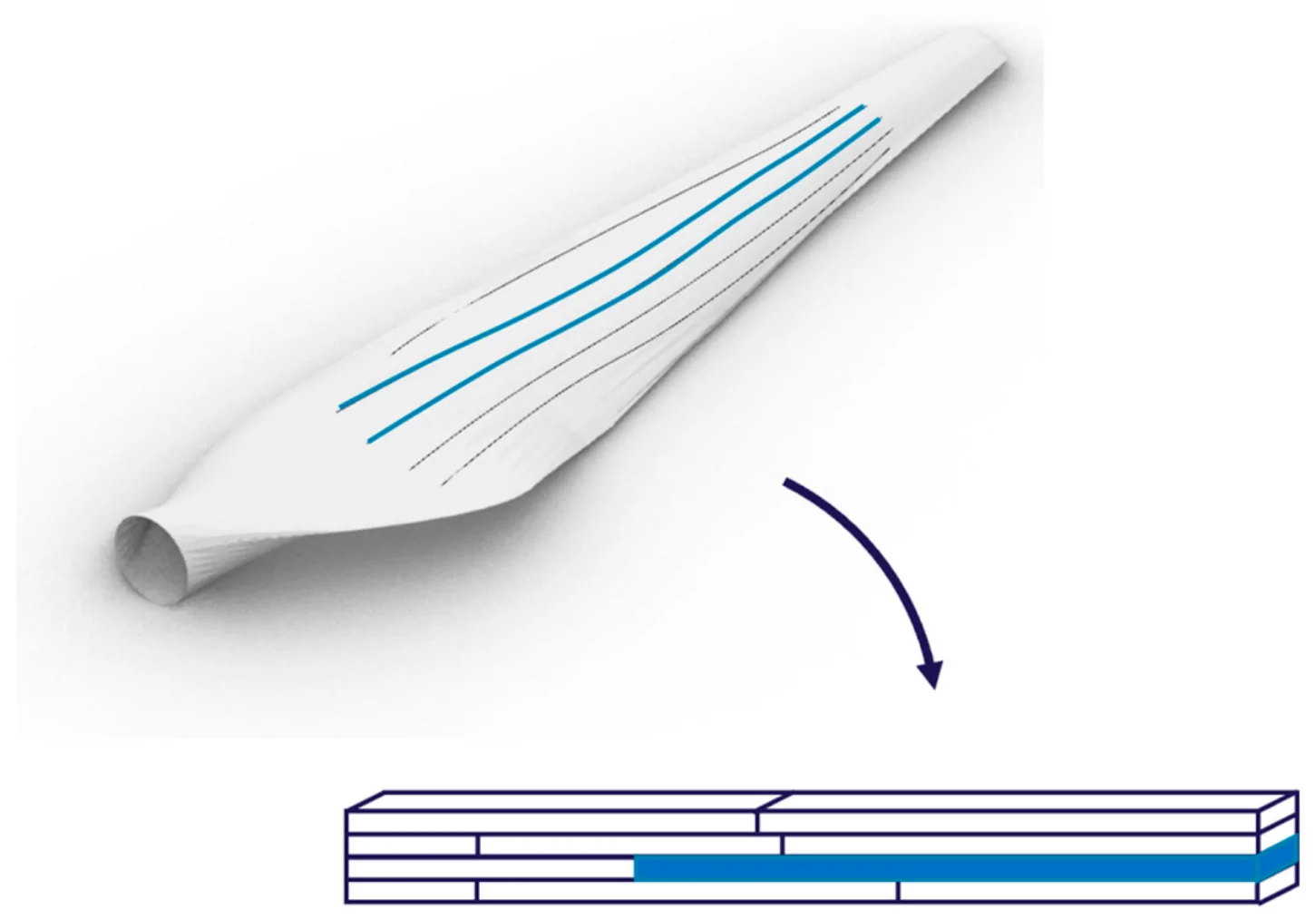
The schematic drawing of how to use a cut-out section from the decommissioned wind turbine blade to form a glued-GFRP-beam. The highlighted blue section represents the cut-out section from the wind turbine blade. Courtesy of Dario Parigi, Aalborg University.

This paper introduces a novel concept for upcycling decommissioned wind turbine blades into structural beams for construction industry. This approach involves cutting out segments of the GFRP shell from the blade and bonding them with adhesive to form a beam (see Figure 3). The resulting beam is constructed using glue-laminated FRP and is hereafter referred to as a GL-FRP beam. To assess the feasibility of the proposed solution, a preliminary study was conducted. Instead of the actual cut-out sections from decommissioned wind turbine blades, commercial GFRP laminates were used and glued together. Adhesive joints were introduced to investigate the behavior of the adhesive interfaces. Then, the flexural performance of the glued beams was further studied using experiments, analytical models, and finite element (FE) simulations. This study provides insight into the feasibility of GL-FRP beams using decommissioned wind turbine blades and supports the development of sustainable end-of-life strategies for wind energy industry.

## 2. Materials and Methods

### 2.1. Material Characterization

#### 2.1.1. Materials

Commercial GFRP laminates were used in this study in place of the blade-derived laminates. The unidirectional GFRP laminates supplied by Fiberline Building Profiles A/S (Fredericia, Denmark) were manufactured through a pultrusion process. This process enables continuous production of composite materials with a constant cross-section and controlled fiber alignment, which contributes to consistent material properties along the longitudinal direction [49]. The GFRP laminates had a uniform cross-section of 100 mm × 10 mm (width × thickness) and were provided in two lengths of 500 mm and 1000 mm. The material properties of the GFRP laminates are presented in Table 2 according to the datasheet provided by the manufacturer Fiberline.

The adhesive used in this study is a two-component methacrylate, Plexus MA310, specifically formulated for bonding thermoplastic, metal, and composites assemblies [51]. According to the datasheet provided by manufacturer ITW Performance Polymers (Shannon, Ireland), the material properties of PlexusMA310 are listed in Table 3.

#### 2.1.2. Material Testing

To verify the material properties provided by manufacturers, the tensile behaviors of GFRP laminates were characterized. Tensile samples of GFRP were prepared according to DS/EN ISO 527-5:2021 [52] with modifications, yielding an effective length of 340 mm, width of 30 mm, and thickness of 10 mm. The tensile test was performed using an MTS testing machine and controlled with a loading rate of 2 mm/min until fracture. To minimize the experimental artifacts, six GFRP tensile specimens were tested. During the experiment, strains were monitored by Rosette strain gauges (Model: FRA-3-350-11, manufactured by Tokyo Measuring Instruments Laboratory Co., Ltd., Tokyo, Japan) at the center of the tensile specimens. The strain gauges feature a gauge length of 3 mm, a resistance of 350 Ω, and a gauge factor of 2.08 ± 1%.

As rosette gauges measured the strains in three directions, namely $\varepsilon_{0}$, $\varepsilon_{45}$, and $\varepsilon_{90}$, strain components could be calculated according to Equation (1).

(1) $$\left\{\begin{array}{c} \varepsilon_{x}=\varepsilon_{0}\\ \varepsilon_{y}=\varepsilon_{90}\\ \gamma_{xy}=2\varepsilon_{45}-(\varepsilon_{0}+\varepsilon_{90}) \end{array}\right.$$

where $\varepsilon_{x}$ and $\varepsilon_{y}$ denote the transverse strain and longitudinal strain, respectively. $\gamma_{xy}$ represents the shear strain. Accordingly, principal strains were calculated as follows:

(2) $$\varepsilon_{1,2}=\frac{\varepsilon_{90}+\varepsilon_{0}}{2}\pm \begin{array}{c} \sqrt{({\frac{\varepsilon_{90}-\varepsilon_{0}}{2})}^{2}+\frac{{\gamma_{xy}}^{2}}{2}}\\ =\frac{\varepsilon_{90}+\varepsilon_{0}}{2}\pm \sqrt{({\frac{\varepsilon_{90}-\varepsilon_{0}}{2})}^{2}+{\left(\varepsilon_{45}-\frac{\varepsilon_{90}+\varepsilon_{0}}{2}\right)}^{2}\;} \end{array}$$

Using the experimental data, tensile strength ($f_{t,y}$), tensile modulus (E), and Poisson’s ratio (ν) were obtained from the experiment and compared to the information from the datasheet provided by the manufacturer. Tensile strength ($f_{t,y}$) was recorded as the stress level when fracture happened. Tensile modulus (E) was calculated based on Equation (3):

(3) $$E=\frac{\sigma }{\varepsilon_{y}}$$

where σ is the effective stress and defined as the ratio of the applied force recorded by the testing machine and the cross-sectional area. In addition, Poisson’s ratio (ν) was calculated using Equation (4). By convention, the negative sign is introduced to yield a positive ratio, assuming axial tension results in transverse contraction:

(4) $$\nu =-\frac{d\varepsilon_{x}}{{d\varepsilon }_{y}}$$

**Figure 4:**
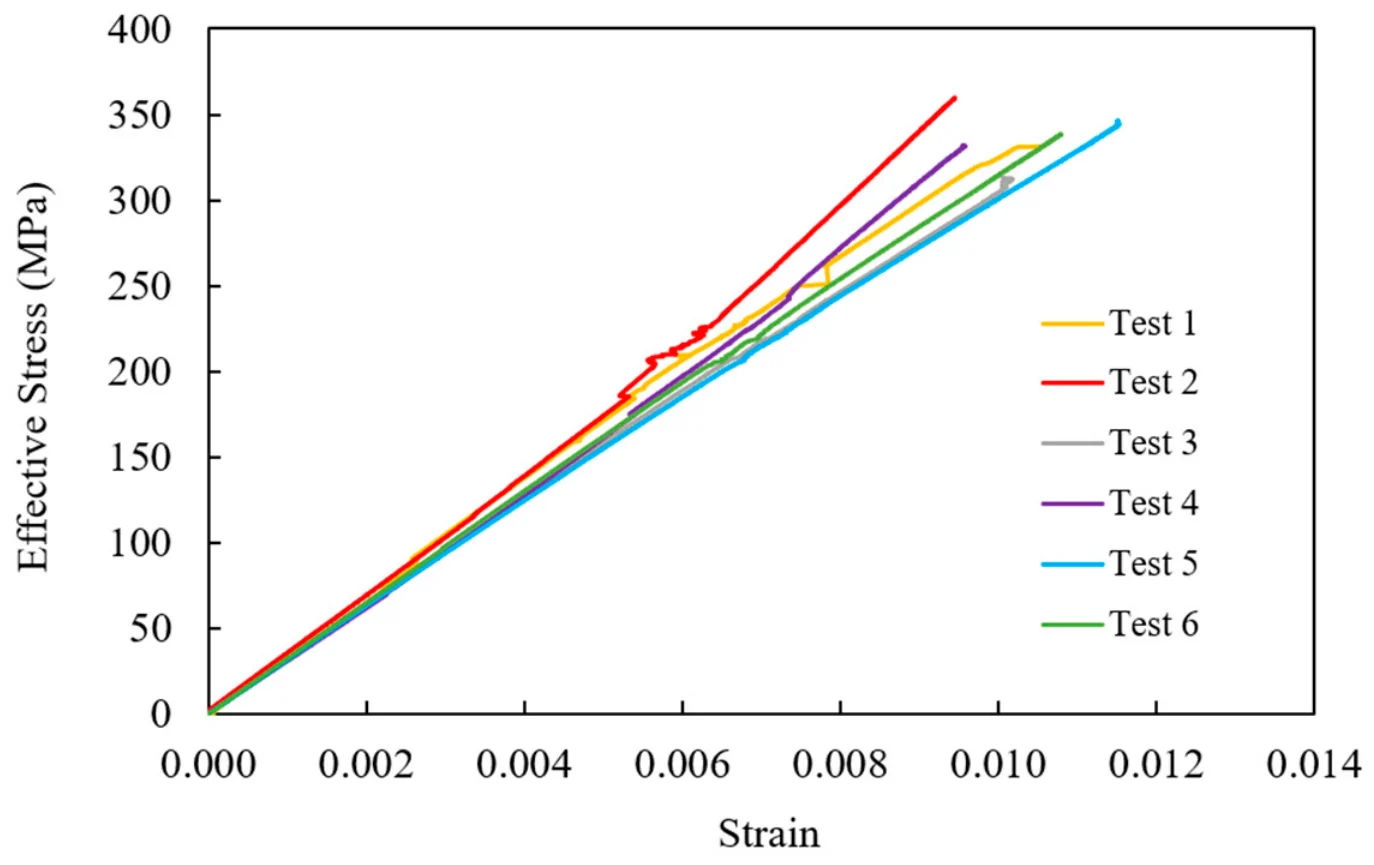
Effective stress strain curves obtained from six tensile tests on GFRP specimens. Curves stopped when a fracture happened.

The effective stress strain curves were obtained from six tests and plotted in Figure 4. It can be observed that all GFRP laminates behaved predominantly in a linear-elastic manner until sudden brittle fracture. Though the polymer matrix could deform slightly nonlinearly, the glass fiber dominated the load-carrying behavior. Hence, the overall stress strain curves were mostly linear until failure, with no obvious plastic deformation.

From the tensile testing results, material properties of GFRP laminates obtained were calculated and summarized in Table 4. Parameters were also listed in the table from the statistical analysis of six tests.

The standard deviation and coefficient of variation (COV) indicate high repeatability of the tensile test results. Therefore, subsequent analyses in this study employed the experimentally obtained GFRP properties. As for the adhesive (Plexus MA310), further analyses adopted its material properties provided by manufacturer (see Table 3).

### 2.2. Experiment

Experiments were carried out to assess the structural mechanism of the adhesive joints and investigate the flexural response of the glued beams.

#### 2.2.1. Adhesive Joints Subjected to Tensile Test

**Figure 5:**
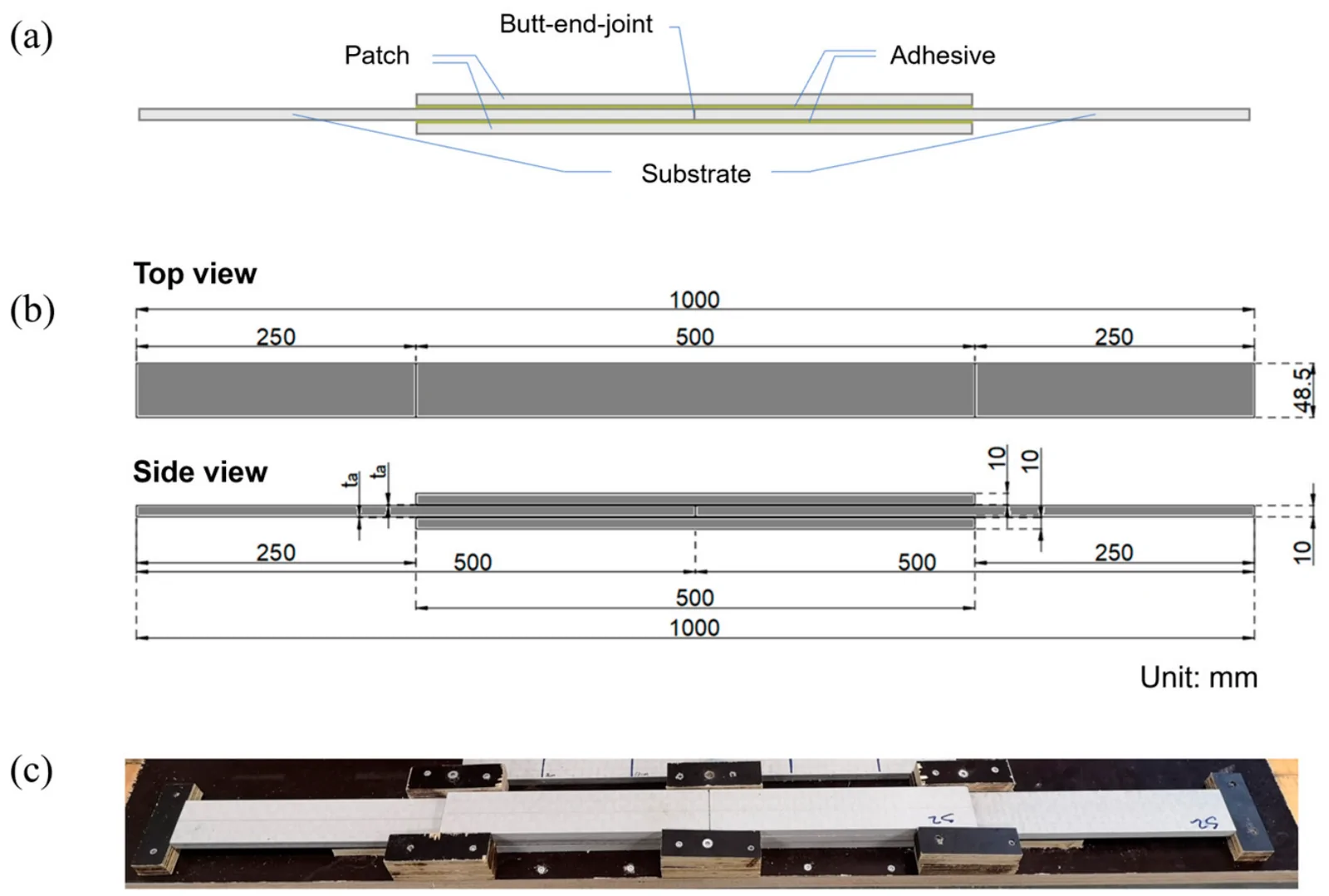
(**a**) Schematic drawing of a double-doubler joint, (**b**) dimensions of the double-doubler joint indicated from both top view and side view, (**c**) and mold for assembling double-doubler joints.

To understand the behavior of adhesive interfaces and facilitate the investigations of glued beams, a double-doubler joint was proposed and tested (see Figure 5a). The double-doubler joint comprised four components: two substrate elements, a butt-end joint connecting the ends of substrates, a patch element on both sides of the substrates, and adhesive layers between the substrate and the patch. The dimensions of the double-doubler joint are shown in Figure 5b. Both substrate and patch elements were made from GFRP laminates, while the adhesive layer was Plexus MA310.

According to the manufacturer, the surface of GFRP laminate should be treated with sandpapers of grit size P60–P100 before gluing to ensure adequate adhesion. Here, laminates with three different surface conditions—untreated, sanded using P80, and sanded with P240 sandpaper—were used to study the influence of surface roughness on the performance of the double-doubler joint. Two adhesive layer thicknesses ($t_{a}$) were applied to examine the influence of glue thickness. Therefore, in total, four different designs of adhesive joints were proposed. Table 5 lists the information of surface treatment of laminates, glue thickness, and number of specimens for four different designs.

To assemble the double-doubler joint, a mold was designed and fabricated to ensure precise alignment of the specimens. The mold comprised a base plate with end and side supports that secured each laminate in position (see Figure 5c). All surfaces of the mold were lined with Teflon to prevent adhesion, and Teflon was also used between the butt-end joint of the substrate elements to avoid unintended bonding. Glue thickness was controlled by inserting 1 mm or 4 mm steel pins between the substrate and patch elements before applying pressure. After adhesive curing, the specimens were demolded and any excess adhesive along the edges was removed to obtain the final geometry.

**Figure 6:**
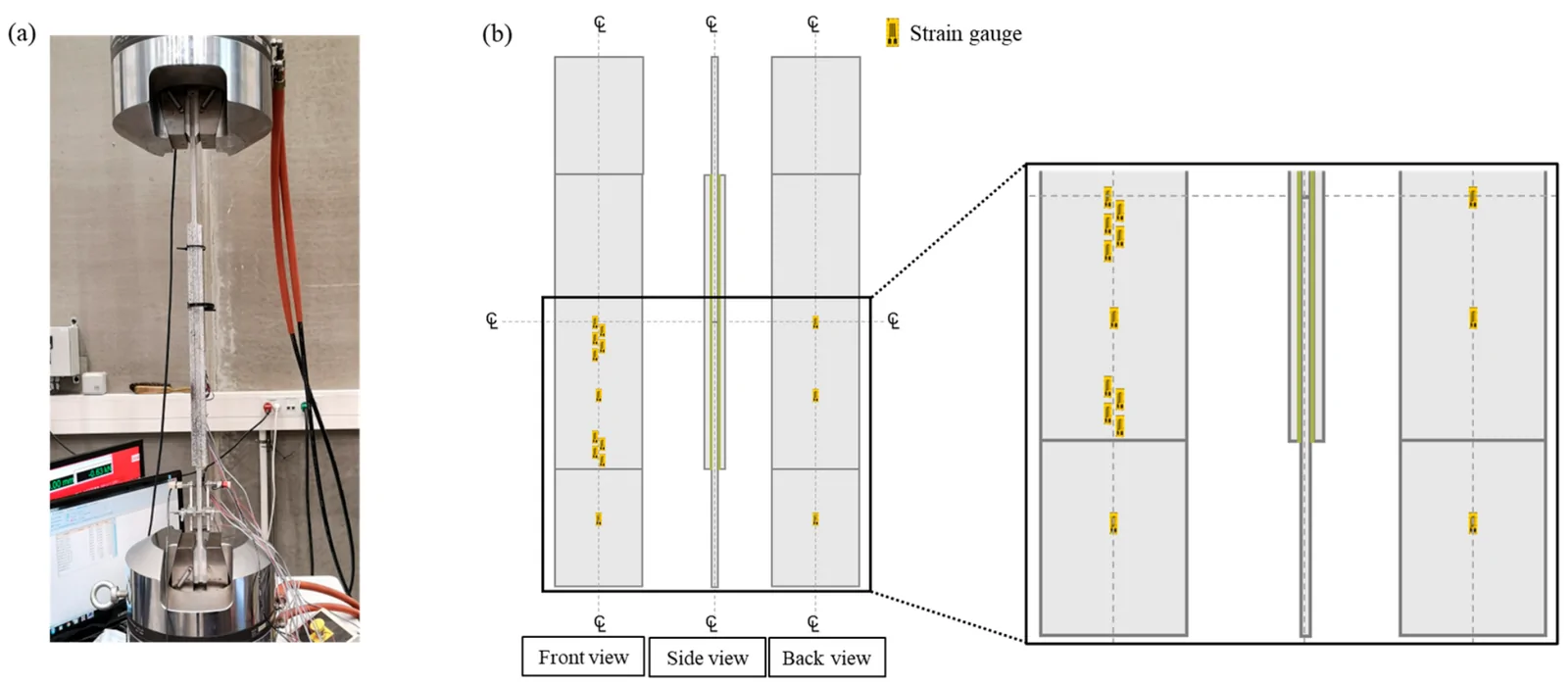
(**a**) Experimental setup for the adhesive joint test using MTS testing machine. (**b**) Strain gauge arrangement for adhesive joint tests with zoom-in pictures showing the accurate locations. Front view and back view in (**b**) correspond to the left and right of specimen in picture (**a**).

Tensile tests were conducted on the samples using an MTS testing machine (see Figure 6a) under a fixed loading speed of 2 mm/min until failure. The design details and number of specimens tested are listed in Table 5. To balance manufacturing complexity, specimen quality control, and statistic reliability, sample size of three specimens per design were selected for the adhesive joint tests. It should be noted that only two valid specimens were evaluated for Design 4 instead of three; this was due to the malfunction of the load measurement during the testing of the initial specimen, leading to unusable data. It is acknowledged that this sample size might have limited statistical robustness. Specimens were monitored by strain gauges for determination of normal stress in the patch and adhesive shear stress in the glue at specific locations. The arrangement of strain gauges is shown in Figure 6b.

A total of 14 strain gauges (SG) were mounted on the GFRP adherends to monitor the adhesive test. Measurements obtained from strain gauges enabled the evaluation of adherend normal stresses and the estimation of shear stresses within the adhesive layer. Two gauges were positioned near the applied load to verify the correlation between the load and the local stress response. Another pair was installed symmetrically about the axial centerline in a region characterized by minimal shear stress. Four gauges were placed in the leading zone of the joint to capture the distribution of adhesive shear stress in this critical region, while another six gauges were mounted along the mid-length of the specimen to obtain the corresponding shear stress.

For each strain measurement, a corresponding stress in the adherends (σ) was calculated. Assuming the material is isotropic, homogeneous and linear elastic, Hooke’s law could be applied:

(5) $$\sigma =\varepsilon E_{GFRP}$$

When two adherends are bonded with a thin adhesive layer and subjected to tensile force, shear stress develops within the adhesive layer to transfer load between them. This load transfer induces a gradient in the normal strain distribution along the adherend surface. Accordingly, adhesive shear stress ($\tau_{a}$) could be determined from the measured strain gradient along the glue line using strain gauges positioned at an interval of ∆x:

(6) $$\tau_{a}\approx \frac{∆\varepsilon_{patch}}{∆x}E_{GFRP}t_{p}$$

where $\frac{∆\varepsilon_{patch}}{∆x}$ is the gradient of the normal strain measured along the patch surface, and $t_{p}$ is the patch thickness.

#### 2.2.2. GL-FRP Beams Under Four-Point Bending Test

**Figure 7:**
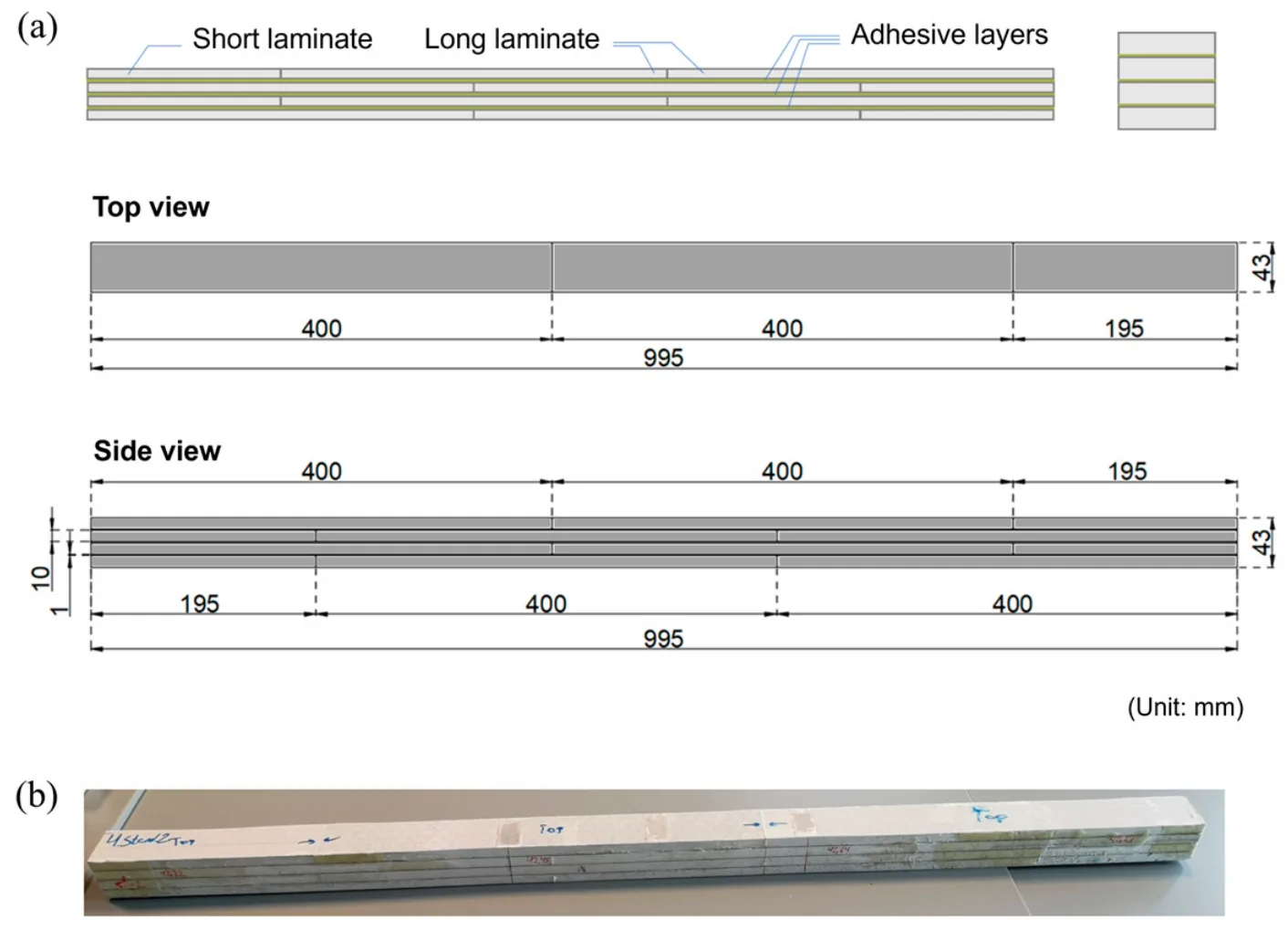
(**a**) Schematic drawings of GL-FRP beam with dimension details; (**b**) picture of an as-fabricated beam specimen.

To further extend the adhesive joint design, GL-FRP beams were proposed. The beam consisted of four GFRP laminate layers, bonded with structural adhesive to form a square cross-section. Laminates were arranged in a staggered pattern, which was similar to traditional bricklaying techniques. Each layer contained one short and two long laminates, with the shorter segment alternating sides between layers to improve load distribution and structural integrity. Schematic drawings of the beam configuration are illustrated in Figure 7a with dimension details.

**Figure 8:**
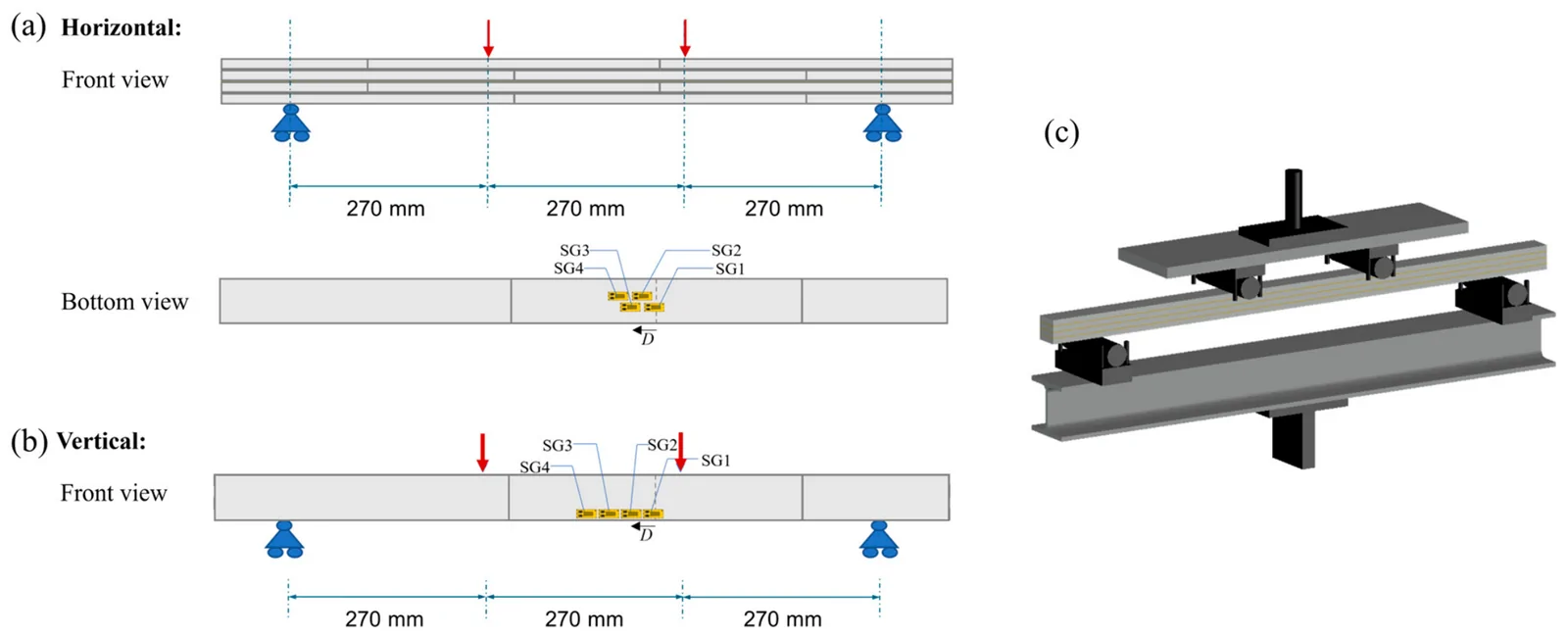
(**a**) Schematic drawings of the four-point bending test on horizontally oriented beam (front view) and locations of strain gauges at the bottom of the beam; (**b**) schematic drawings of the four-point bending test on vertically positioned beam (front view) and locations of strain gauges on the side of the beam; (**c**) 3D drawing of the four-point bending setup. The red downwards arrows represent the point loads.

Similar to the adhesive joint, a mold was crafted to assist assemble the glued beam. The mold consisted of end and side supports, with side supports positioned at each butt-end joint. All mold surfaces were lined with Teflon to prevent adhesion. Beam assembly followed the same procedure as the adhesive joint, and an as-fabricated GL-FRP beam is shown in Figure 7b. Four-point bending tests were conducted to study the flexural behavior of the beams, modified based on DS/EN ISO 14125:1998 [53]. Beams were tested in two configurations: laminates arranged horizontally (lying) and vertically (standing) as illustrated in Figure 8a,b. All beams were manufactured through an identical process and positioned accordingly to achieve the desired orientation for testing.

In the test setup, two supports were placed 810 mm from each other. Two point-loads were assigned at the trisection points, which were 270 mm apart from each other and from their adjacent supports (see Figure 8a,b). To perform the four-point bending test, a test rig was designed (see Figure 8c). It consisted of the supports at the bottom and the loading at the top. The bottom part where supports stand was made of a HEA100 beam, which had a relatively higher bending stiffness compared to the GL-FRP beam to avoid deformation in the setup of any importance. Above the specimen, a thick steel plate was used to mount the two point-loads. The beams were subjected to a displacement-controlled loading at 2 mm/min. For each beam configuration, three specimens were tested, and their corresponding force-displace curves were obtained.

Moreover, strain gauges were installed to monitor the beam deformation during testing (see Figure 8a,b). For the horizontal configuration, gauges were mounted along the bottom surface (see Figure 8a)—one centered beneath the butt-end joint and three others positioned 10 mm, 20 mm, and 30 mm away along the length. For the vertically oriented beam (see Figure 8b), strain gauges were attached to the side surface at a height of 3 mm and spaced 20 mm apart. Again, the rightmost gauge sat at the laminate mid-line that was beneath the butt-end joint. The strain gauge measurements were used to estimate the corresponding stress in the glued beams.

### 2.3. Analytical Solutions

#### 2.3.1. Direct Linear-Elastic Solutions for Adhesive Joints

A direct linear-elastic model [54] was adopted to understand the behavior of the adhesively bonded joint proposed in this study. The model provides analytical solutions to adhesive shear stress distribution and adherend normal stress distribution in a symmetric adhesive joint. Compared to other models that require high computational cost and time, this one-dimensional analysis technique provides an easy-to-use avenue for investigating stress distribution of adhesive joints. This approach is built based on the following assumptions:

(a) The adhesive joint behaves in a linear elastic manner.
(b) The geometry of the adhesive joint is symmetric.
(c) Thicknesses of both the substrate elements and patch are uniform.

**Figure 9:**
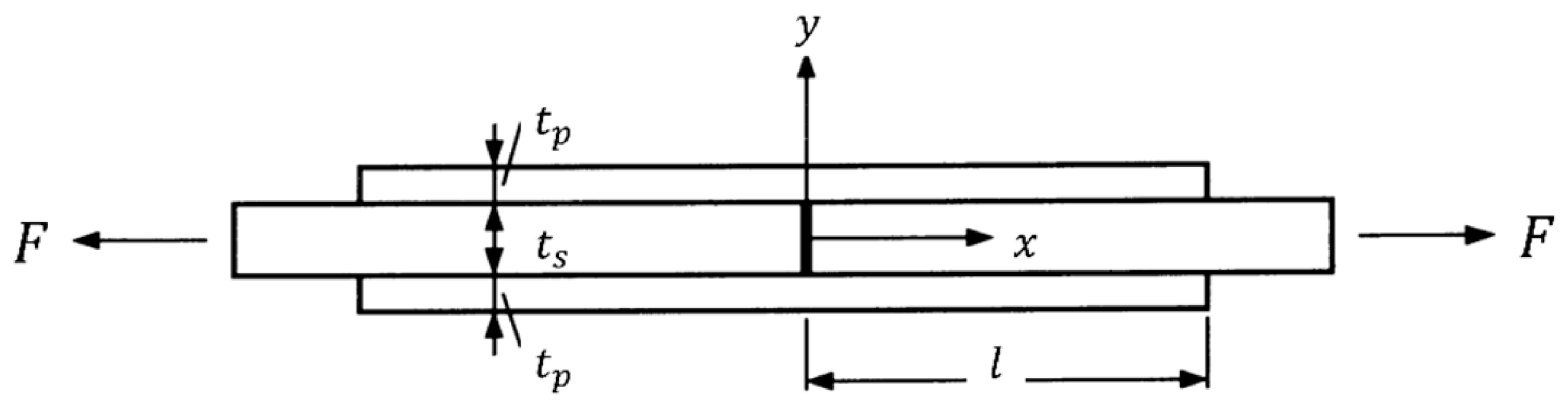
Double-doubler joint with uniform substrate and patch thickness. It should be noted that, in this study, the thickness of the patch ($t_{p}$) and the thickness of the substrate ($t_{s}$) are the same. It is later referred to as $t_{ps}$ in the equations.

The adhesive joint proposed in this study is a double-doubler joint (see Figure 9), assuming no tensile force could transfer at the butt-end joint. Since the same material (GFRP laminate) was used for both patch and substrate elements, the solutions could be further simplified based on the original expressions [54]. Equations (7)–(9) express the linear-elastic solutions to adhesive shear stress ($\tau_{a}$), normal stress in the substrate elements ($\sigma_{s}$), and normal stress in the patch ($\sigma_{p}$):

(7) $$\tau_{a}\left(x\geq 0\right)=\frac{\lambda }{2}\frac{F}{3}\left(\sinh\left(\lambda x\right)-\frac{\cosh\left(\lambda x\right)}{\tanh\left(\lambda l\right)}\right)-\lambda \frac{F}{3}\frac{\cosh\left(\lambda x\right)}{\sinh\left(\lambda l\right)}$$

(8) $$\sigma_{s}\left(x\geq 0\right)=\frac{F}{3t_{ps}}\left(1-\cosh\left(\lambda x\right)+\frac{\sinh\left(\lambda x\right)}{\tanh\left(\lambda l\right)}\right)+2\frac{F}{3t_{ps}}\frac{\sinh\left(\lambda x\right)}{\sinh\left(\lambda l\right)}$$

(9) $$\sigma_{p}\left(x\geq 0\right)=\frac{1}{2}\frac{F}{3t_{ps}}\left(\cosh\left(\lambda x\right)-\frac{\sinh\left(\lambda x\right)}{\tanh\left(\lambda l\right)}\right)+\frac{F}{3t_{ps}}(1-\frac{\sinh\left(\lambda x\right)}{\sinh\left(\lambda l\right)})$$

where F is the applied force per unit length on the double-doubler joint, λ denotes the elastic shear stress distribution parameter, x is the axial coordinate of the joint, and $t_{ps}$ is the thickness of the patch and substrate element in this study.

It should be noted that shear-lag and peel stress are not considered in this one-dimensional solution. The peel stresses were not treated here. However, shear lag has been shown to have significant importance, especially for adherends with a low shear modulus such as GFRP composite. Therefore, the elastic shear stress distribution factor, λ, was corrected [54]. The shear stress distribution through the adherends can be assumed linear due to the relatively thin thickness of the adherends as well as the zero shear stress at free surfaces. To account for shear lag [54], the elastic shear stress distribution factor, λ, was written as:

(10) $$\lambda =\sqrt{\frac{\frac{3}{E_{GFRP}t_{ps}}}{\frac{5}{8}\frac{t_{ps}}{G_{GFRP}}+\frac{t_{a}}{G_{a}}}}$$

In addition, the correction of λ, another modification was also included here. Equation (9) describes the normal stress on the patch surface that directly contacts the glue in the adhesive joint. However, during the adhesive joint test, the strain gauges monitored the strain on the patch surface facing away from the glue. As the force in the patch element was transferred through shear in the adhesive joint, it could be considered as an eccentric force acting on the patch element. The eccentricity then resulted in a moment inducing compression in the outer surface of the patch. To take this into account, the direct linear-elastic solution was therefore modified to be comparable to the experimental results regarding the normal stress distribution in the patch. The eccentric load ($q_{a}\left(x\right)$) is calculated based on the shear stress and the cross-sectional area ($A_{a}$) of the glue:

(11) $$q_{a}\left(x\right)=\tau_{a}\left(x\right)A_{a}$$

Hence, the moment that was generated from the eccentric load and acting on the patch was derived:

(12) $$M_{p}\left(x\right)=q_{a}\left(x\right)e=q_{a}\left(x\right)\frac{t_{ps}}{2}$$

where e denotes eccentricity and equals to half of the patch thickness ($\frac{t_{ps}}{2}$). Using the relationship between stress and moment, the compressive stress on the outer surface of patch element could be determined by Equation (13):

(13) $$\sigma_{p,c}\left(x\right)=\frac{M_{p}\left(x\right)y}{I_{p}}=\frac{M_{p}\left(x\right)\frac{t_{ps}}{2}}{\frac{1}{12}t_{ps}^{3}b}$$

Hence, the normal stress on the outer surface of patch ($\sigma_{p,outer}$) was calculated by subtracting the compressive stress from the stress determined using the direct linear-elastic solution:

(14) $$\sigma_{p,outer}\left(x\right)=\sigma_{p}-\sigma_{p,c}$$

#### 2.3.2. Analytical Predictions for GL-FRP Beams

**Figure 10:**
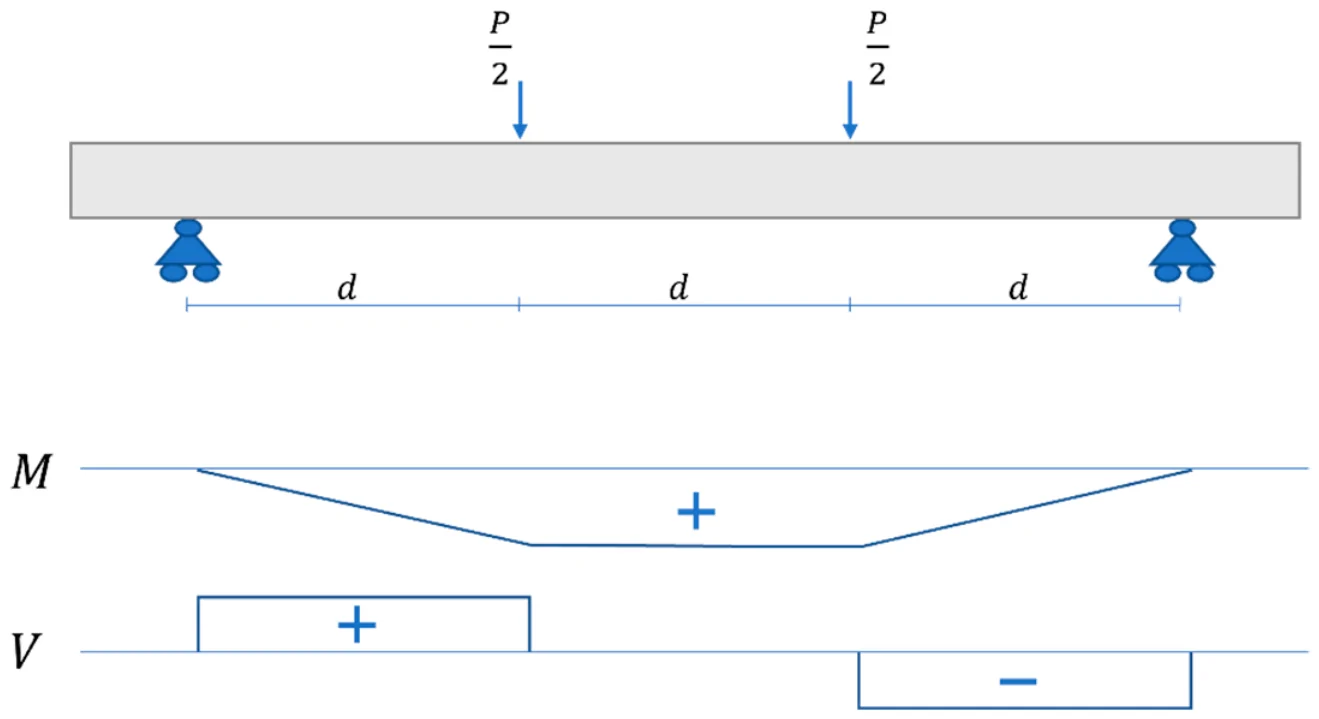
Bending moment diagram and shear force diagram for beam subjected to four-point bending.

Stress distributions in GL-FRP beams were also predicted analytically at the locations where they were captured in the experiment. For beams under four-point bending, the bending moment diagram and shear force diagram are shown in Figure 10. The region between the point loads experienced a constant bending moment and zero shear force, while the segments between each support and the adjacent load were subjected to a linearly varying moment and a constant shear force. The cross-sectional forces can be determined by equilibrium equations.

The bending moment was assumed to vary linearly across the beam height. Normal stress was evaluated by dividing the cross-section into compressive and tensile zones, with stress at any point calculated using Equation (15).

(15) $$\sigma =\frac{My}{I}$$

where M is the bending moment, y is the distance from the neutral axis, and I is the moment of inertia.

**Figure 11:**
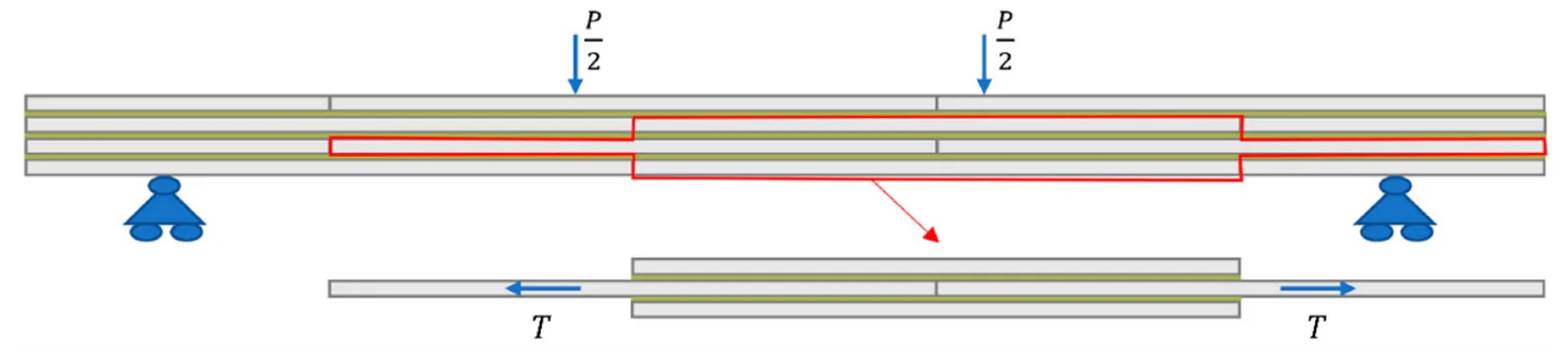
Double-doubler joints in the beam positioned horizontally. The double-doubler joint highlighted here was used to analyze the normal stress distribution in the bottom laminate and the shear stress distribution in the bottom adhesive layer. The tensile forces resulted from the bending are shown as T.

For beams with laminates lying horizontally, several double-doubler joints could be identified as shown in Figure 11. The bottom joint, which is highlighted in the figure, was used for predicting normal stress in the bottom long laminate and shear stress in the bottom glue line to compare with experimental and numerical results.

Normal stress in the bottom laminate was developed from two loadings: the global bending moment and the resulting tensile force T acting on the substrate element (see Figure 11). From the beam geometry, stress state of the bottom double-doubler joint was identified. The top patch element was under compression, whereas the substrate elements and the bottom patch were subjected to tension. The resulting tensile force T can subsequently be derived by multiplying the normal stress obtained from Equation (15) by the substrate element’s cross-sectional area. The corresponding normal stress developed in the laminate was then determined according to the direct linear-elastic solution for double-doubler joint. By combining the contributions from global bending moment and the resulting tensile force, the expression for the normal stress distribution in the bottom laminate of the glued beam is written as:

(16) $$\begin{array}{cc} \sigma_{laminate}\left(x\geq 0\right) & =\frac{M(x)}{I}\times \frac{t}{2}+\frac{1}{2}\frac{T}{3t_{ps}}\left(\cosh\left(\lambda x\right)-\frac{\sinh\left(\lambda x\right)}{\tanh\left(\lambda l\right)}\right)\\ & +\frac{T}{3t_{ps}}\left(1-\frac{\sinh\left(\lambda x\right)}{\sinh\left(\lambda l\right)}\right)-\frac{M_{p}\left(x\right)\frac{t_{ps}}{2}}{\frac{1}{12}t_{ps}^{3}b} \end{array}$$

where t and b denote the height and the width of the beam. Based on the bending moment diagram, the resulting tensile force T remained constant between the point loads and decreased from each point load toward the adjacent support. Therefore, the shear stress distribution along the bottom adhesive layer was analyzed separately for these two regions. Within the constant moment zone, tension between the laminates was transferred through shear stress. Thus, shear stress distribution was determined by Equation (7). However, between each point load and the adjacent support, shear stress was not only contributed by stress transfer but also the shear force. The contribution from the shear force ($\tau_{V}$) was calculated using beam theory expression:

(17) $$\tau_{V}=\frac{VQ}{Ib}$$

where V is the shear force at the cross-section, and Q is the first moment of area. Therefore, shear stress distribution within the region where the shear force was not zero was calculated by combining Equations (7) and (17). To summarize, adhesive shear stress in the bottom glue could be written as:

(18) $$\tau_{a}=\frac{\lambda }{2}\frac{T}{3}\left(\sinh\left(\lambda x\right)-\frac{\cosh\left(\lambda x\right)}{\tanh\left(\lambda l\right)}\right)-\lambda \frac{T}{3}\frac{\cosh\left(\lambda x\right)}{\sinh\left(\lambda l\right)}+\left\{\begin{array}{c} 0,\;\;\;\;\;\;constant\;moment\\ \frac{VQ}{Ib},\;\;\;\;\;\;\;constant\;shear \end{array}\right.$$

Different from the horizontal configuration, the vertically oriented beam was assumed to be subjected to pure bending. Hence, the double-doubler joint equations were not applicable here. Instead, normal stress in the laminate was determined by Equation (15), and the corresponding adhesive shear stress was calculated using Equation (17).

### 2.4. Finite Element Simulations

Finite element (FE) models were developed using Abaqus/Explicit 2020 (Dassault Systems SIMULIA Corp.) to simulate the responses of the adhesive joints under tension as well as GL-FRP beams subjected to bending. The constitutive behaviors of GFRP and the adhesive were modeled as elastic, and their material properties were obtained from the material characterization and datasheet, respectively.

A three-dimensional FE model of the proposed adhesive joint was developed to simulate the tensile test. The dimensions of the FE models for the adhesive joints were shown in Figure 5. The GFRP adherends, including two patches and two substrate elements, were modeled as linear elastic solids, with a Young’s modulus of 32160 MPa, a Poisson’s ratio of 0.32, and a density of 1.8 g/cm^3^. The adhesive layers were assigned linear elastic properties with a Young’s modulus value of 1100 MPa, a Poisson’s ratio of 0.3, and a density is 0.99 g/cm^3^. Tie constraints were defined between the contacting surfaces of GFRP laminates and adhesive layers. The left end surface of the substrate was constrained in all translational degrees of freedom, while the right end surface of the substrate was restrained laterally but allowed to move in the longitudinal direction. A prescribed axial displacement was then applied to the right substrate end to simulate tensile loading, generating an axial stress of approximately 43.57 MPa in the substrate.

**Figure 12:**
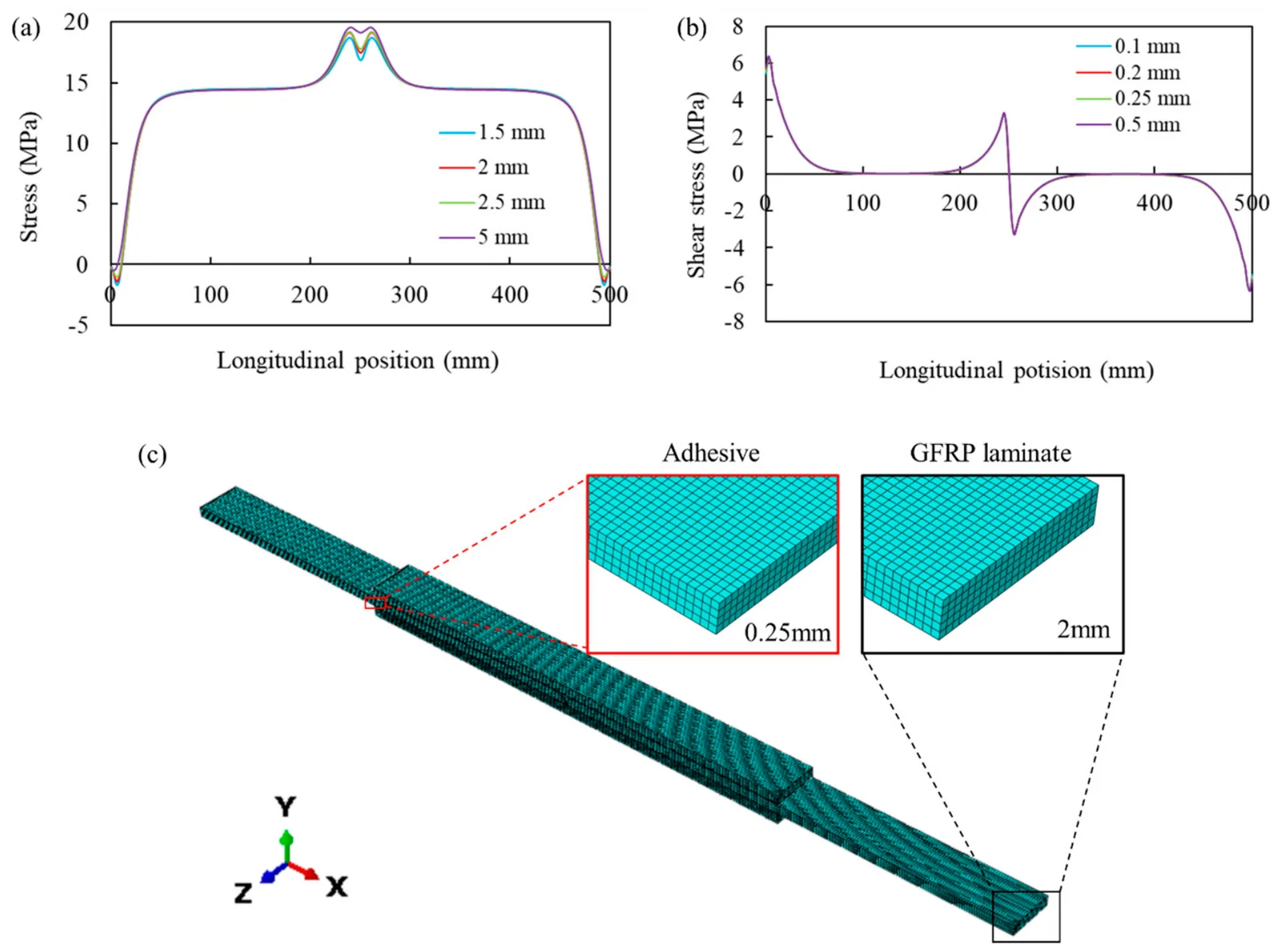
(**a**) Mesh size convergence studies for GFRP laminates, using the normal stress distribution in the top patch as a measuring property; (**b**) mesh size convergence studies for adhesive, using the shear stress distribution in the adhesive as a measuring property; (**c**) meshed 3D adhesive joint (*t* = 1 mm), showing mesh details for GFRP laminates and adhesive layers. In the magnified meshes, the hexahedral mesh of the GFRP laminates has a side length of 2 mm, and the hexahedral mesh of the adhesive has a side length of 0.25 mm.

All parts were discretized with eight-node, linear hexahedral solid (C3D8R) elements. To optimize computational efficiency and accuracy, a mesh size convergence study was conducted first on the adhesive structure with a glue thickness of 1 mm. Four different mesh sizes (1.5 mm, 2 mm, 2.5 mm, and 5 mm) were evaluated for the patches and substrate elements, while another four different mesh sizes (0.1 mm, 0.2 mm, 0.25 mm, and 0.5 mm) were assessed for the adhesive layers. Results (see Figure 12a,b) show that 2 mm and 0.25 mm were the optimal mesh sizes to capture the response of the GFRP laminates and the adhesive, respectively, to guarantee desired accuracy and reduce computational cost at the same time.

Three-dimensional FE models of GL-FRP beams were also developed to simulate the four-point bending tests. The geometric dimensions of the beam models are illustrated in Figure 7. The material properties were identical to those used in the FE model of the adhesive joints. Six tie constraints were assigned at the interfaces between GFRP laminates and three adhesive layers. Boundary conditions were applied by constraining the nodes at the support locations in two directions while allowing free axial movement. Similarly, the nodes corresponding to the loading points were constrained in two directions and allowed translation in the z-direction. In the first analysis step, prescribed displacements of 2 mm and 11 mm in the z-direction were applied to loading nodes for the horizontally and vertically oriented beam, respectively. All parts were discretized using eight-node, linear hexahedral solid (C3D8R) elements.

During beam assembly, Teflon tape was placed between adjacent laminates within the same layer to ideally prevent direct stress transfer. However, the possibility of glue infiltration could result in unintended bonding between these laminates. To account for this uncertainty and enable a more realistic evaluation, two scenarios were considered in numerical simulations: (i) no direct stress transfer, (ii) perfect bonding between adjacent laminates within the same layer. Accordingly, two types of interfacial interactions were defined—no interaction and tie constraints between laminates. Results for both cases are presented in the subsequent analysis and compared with the experimental and analytical results.

## 3. Results

### 3.1. Experimental Results

#### 3.1.1. Adhesive Joint Test Results

**Figure 13:**
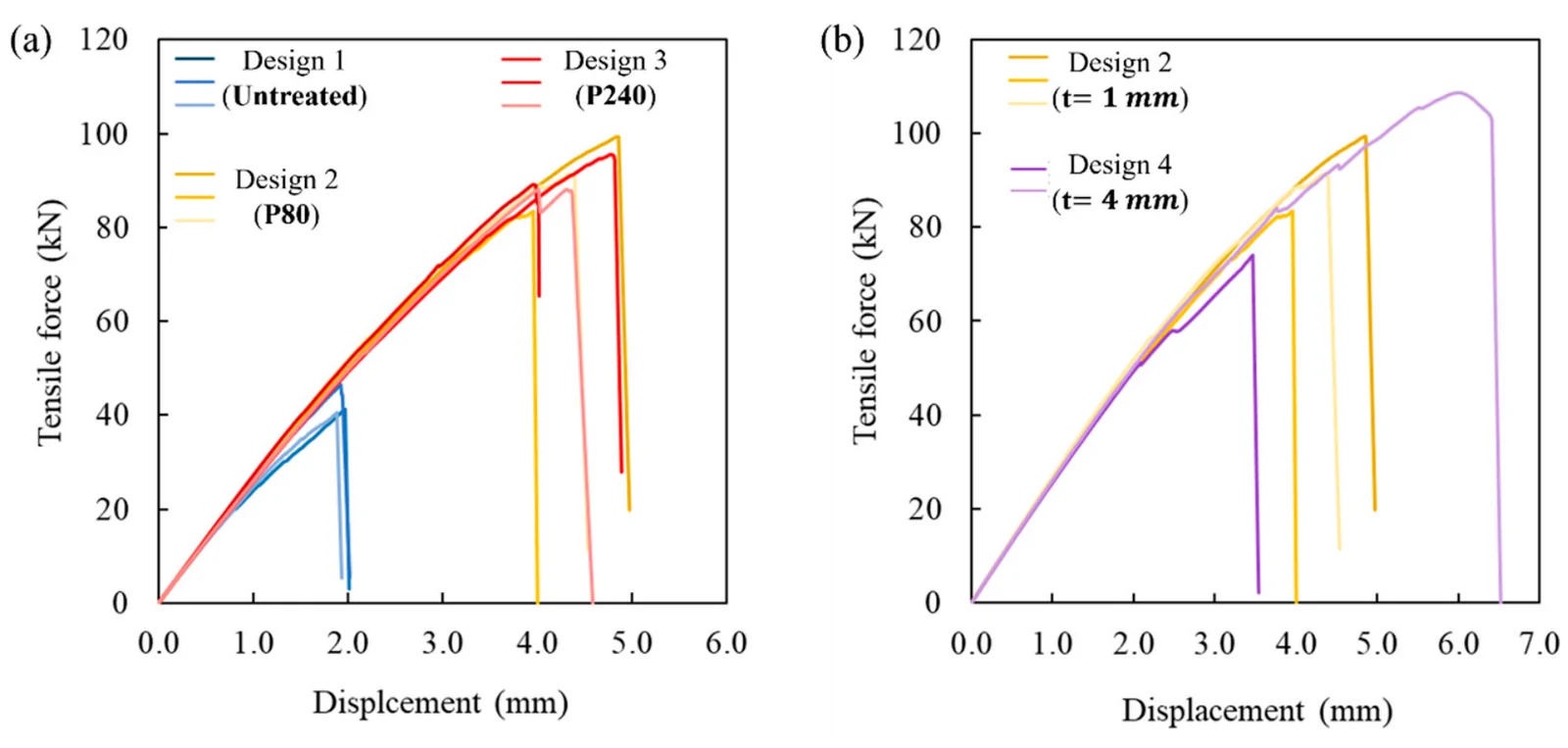
Force displacement curves obtained from tensile experiment of adhesive joints to compare the mechanical response of (**a**) different surface roughness of GFRP laminates; (**b**) different glue thickness.

To investigate the mechanical performance of four different designs, Figure 13 shows the force-displacement curves obtained from tensile testing. All curves exhibited a linear region, indicating that specimens underwent an elastic deformation phase. As shown in Figure 13a, the adhesive joints without laminate surface treatment (Design 1) failed at approximately 40 kN. In contrast, the specimens with abraded surfaces (Design 2 and Design 3) exhibited higher load-bearing capacity with failure occurring at over 80 kN. The results from Design 1 were consistent, whereas noticeable variations were observed among the specimens from Design 2 and Design 3. These discrepancies were considered reasonable, as the actual adhesive thickness of the specimens exhibited comparable differences (ranging from 0.61 mm to 0.97 mm for Design 2 and from 0.62 mm to 0.81 mm for Design 3). Although the glue thickness was designed to be 1 mm, the measured value deviated slightly due to sample preparation. The untreated joints failed by slip at the adhesive-substrate interface, whereas the sanded specimens failed due to delamination. The delamination initiated at one end of the patch elements and propagated along the adhesive bond line. These results suggest that mechanical roughening of the laminate surfaces could largely improve interfacial adhesion. However, the variation in surface roughness produced by P80 and P240 sandpapers had a negligible effect, as both designs yielded comparable ultimate strength and same failure mechanism.

Furthermore, Figure 13b compares the force displacement curves of adhesive joints with different glue thickness. As explained earlier, the specimens with 1 mm glue (Design 2) showed comparable behaviors with load-bearing capacities varying between 80 kN and 100 kN. However, results for adhesive joints with glue thickness of 4 mm (Design 4) were not consistent. One specimen exhibited higher load-bearing capacity than joints with 1 mm glue and failed at approximately 110 kN. This increase of ultimate load was due to a more uniform stress distribution within the joint with a thicker adhesive layer. Nevertheless, the other specimen failed at a much lower force (70 kN). This could be attributed to a premature failure initiated at overlapping edges, preventing full mobilization of cohesive strength.

**Figure 14:**
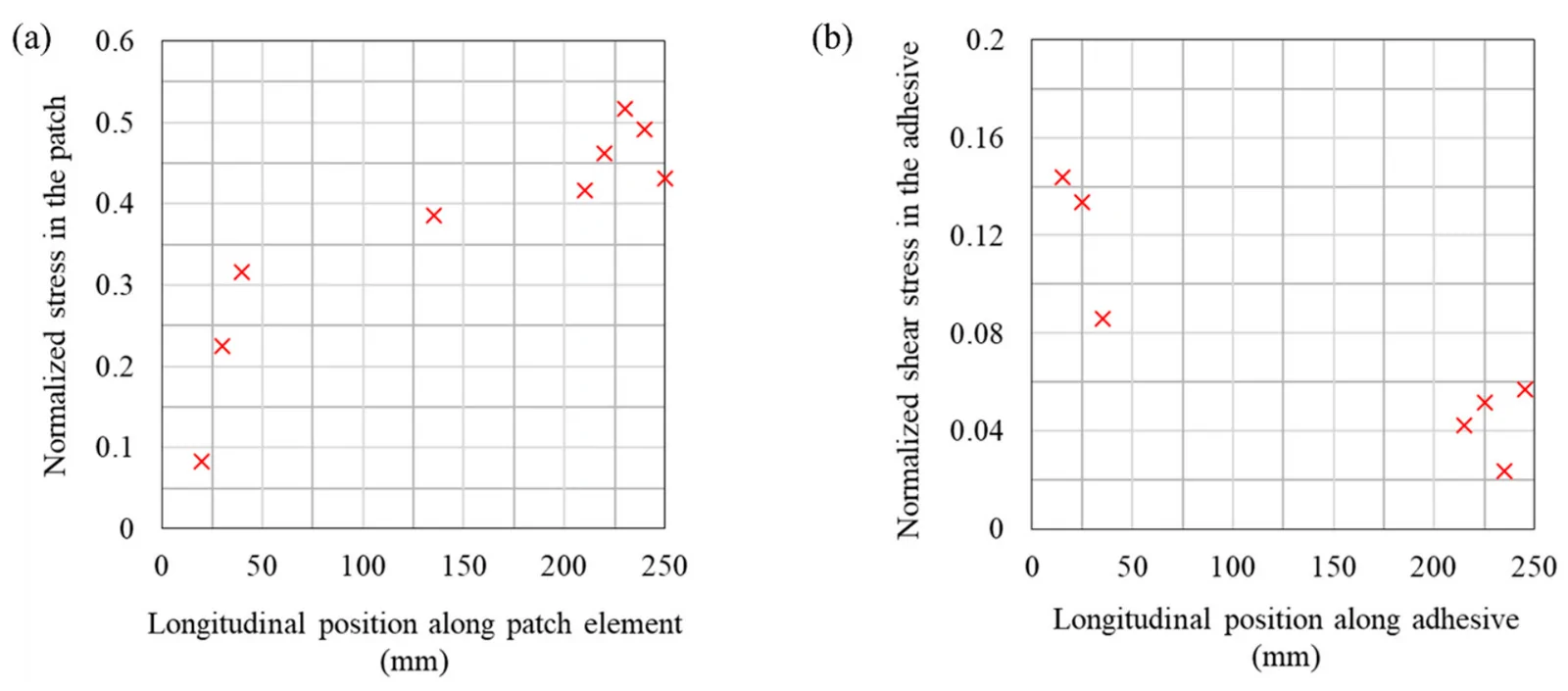
Normalized stress calculated from strain gauge measurements for a specimen of Design 2 (sanded by P80 sandpaper, t=1 mm), including (**a**) normalized normal stress in the patch and (**b**) normalized shear stress in the adhesive.

Based on the strain gauge measurements, stress in the patch and adhesive were calculated and then normalized by the stress in the substrate. Normalization eliminated dependence on load level and specimen geometry, facilitating comparisons among experimental results, analytical predictions, and FE simulations. Figure 14 presents the normalized normal stress in the patch and normalized shear stress in the adhesive for a specimen from Design 2 (sanded by P80 sandpaper, t=1 mm).

#### 3.1.2. Four-Point Bending Test Results

**Figure 15:**
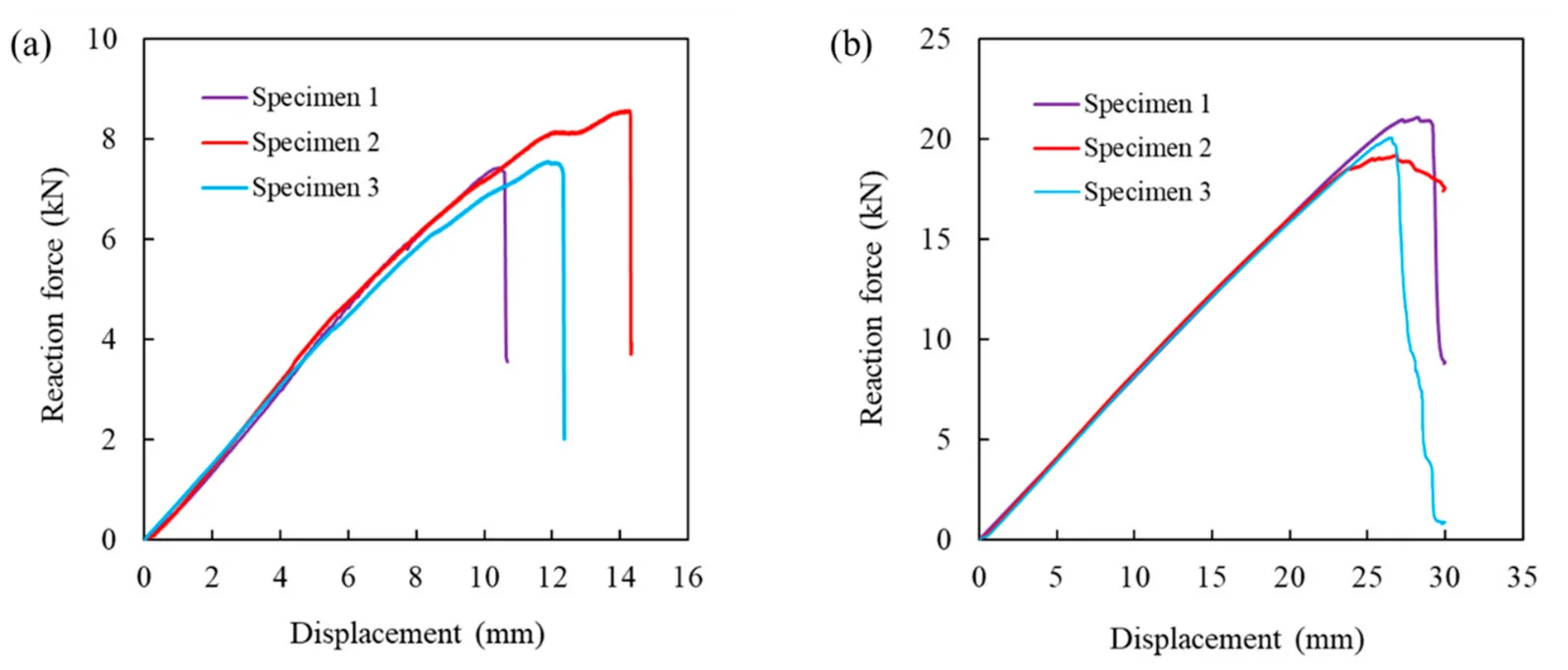
Force displacement curves obtained from the testing machine: (**a**) for the horizontally positioned beam, and (**b**) the vertically positioned beam.

The force-displacement curves from the bending tests were plotted in Figure 15. It should be noted that the x-axis ‘displacement’ refers to the axial displacement of the point loads and recorded by the testing machine. A linear region could be observed from all curves, which indicates elastic behaviors of the beams regardless of their orientation. In addition, the failure loads of the vertically oriented beams were more than twice those of the horizontally positioned beams. This suggests that the glued beams obtained higher load-bearing capacity when the laminates were oriented vertically rather than horizontally.

**Figure 16:**
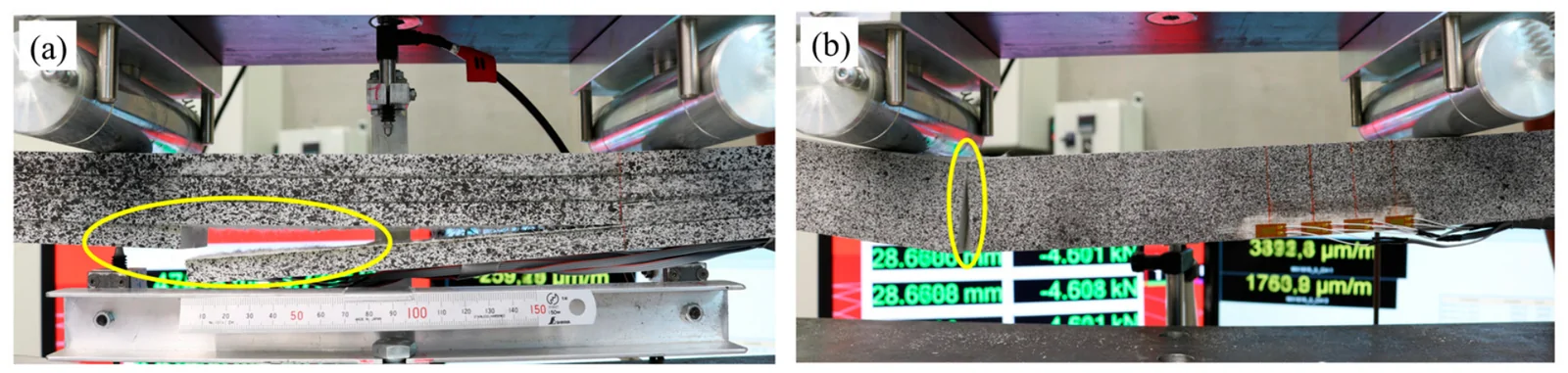
Failure mechanisms (highlighted by the yellow circles) of the glued beam that was positioned (**a**) with laminates lying horizontally, and (**b**) with laminates lying vertically.

**Figure 17:**
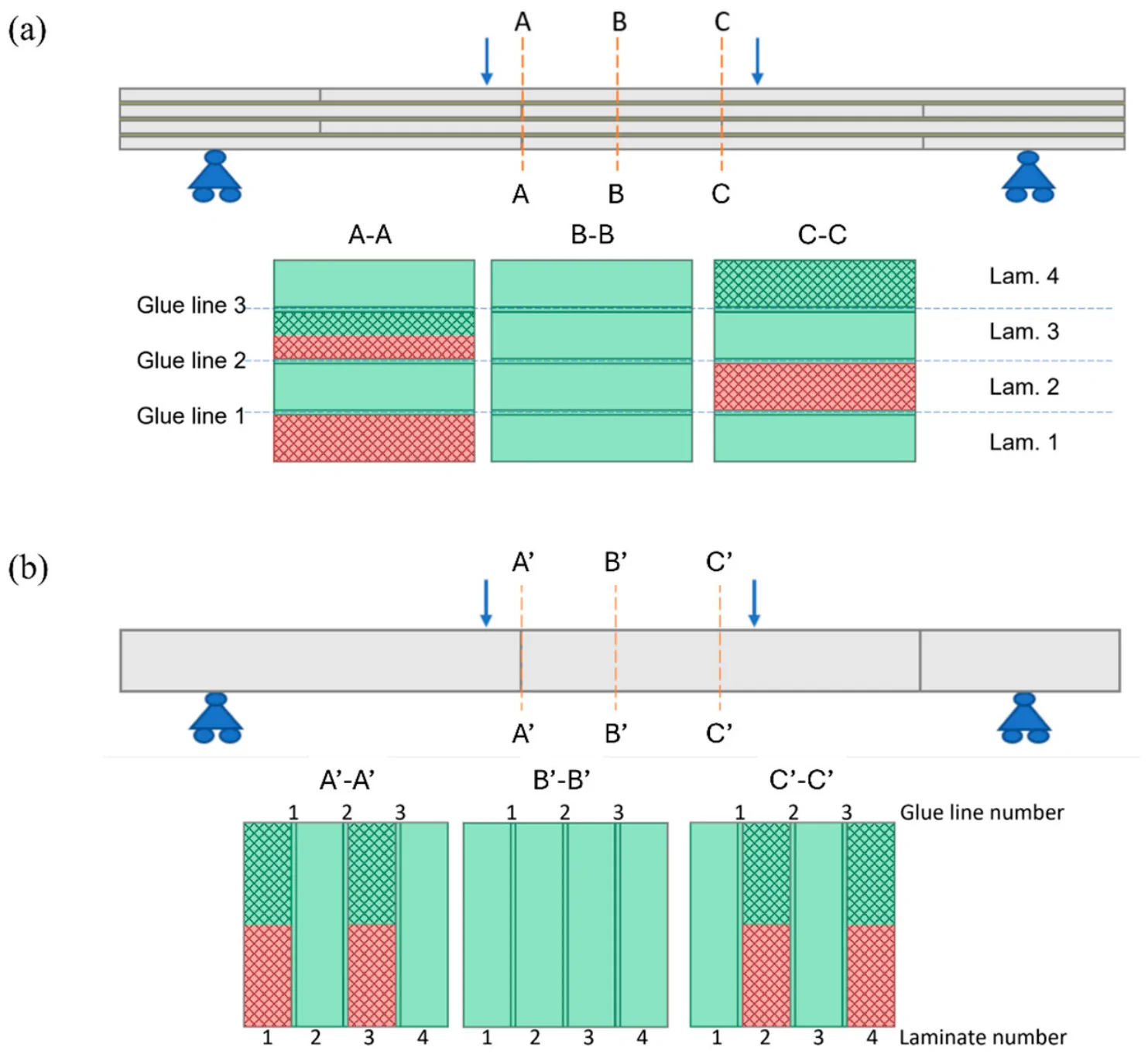
Cross-sections of the GL-FRP beam for determining the moment of inertia: (**a**) with laminates oriented horizontally, (**b**) with laminates oriented vertically. The hatched area represents butt-end joint, green indicates active zone, and red indicates inactive zone. Downwards arrows represent point loads.

The observed failure mechanisms are illustrated and highlighted in Figure 16. In the horizontal configuration, failure was caused by delamination. This failure mechanism was consistent with what was observed in the adhesive joint tests. Differently, the vertically oriented beams failed due to tensile rupture from the lower part of the beams. The locations of failure could be explained by the moment of inertia. As demonstrated in Figure 17, three types of cross-sections were identified along the beam for both horizontal and vertical configurations.

For the horizontally positioned beam, it is clear to see in Figure 17a that the weakest cross-section is A−A. Therefore, the failure initiated from cross-section A−A in the experiment. More specifically, the beam failed from delamination at the bottom of laminate 2 which was consistent with the experimental observation in Figure 16a. For the vertical configuration, the moments of inertia of cross-section A’−A’ and C’−C’ are equal. Failure could initiate at either cross-section, depending on fabrication quality of the beam. This explains the tensile rupture at the lower part of the beam in Figure 16b.

The reason why vertically oriented beams have higher load-bearing capacity also lies in the moment of inertia of the critical cross-section of the beam. The weakest cross-section A−A of horizontal configuration yields a smaller moment of inertia ($113,300\;{\mathrm{mm}}^{4}$) compared to the weakest cross-sections A’−A’ and C’−C’ of vertical configuration ($182,900\;{\mathrm{mm}}^{4}$). Therefore, the stress induced at the critical cross-section in horizontally positioned beam was higher than that in vertically positioned beam.

**Figure 18:**
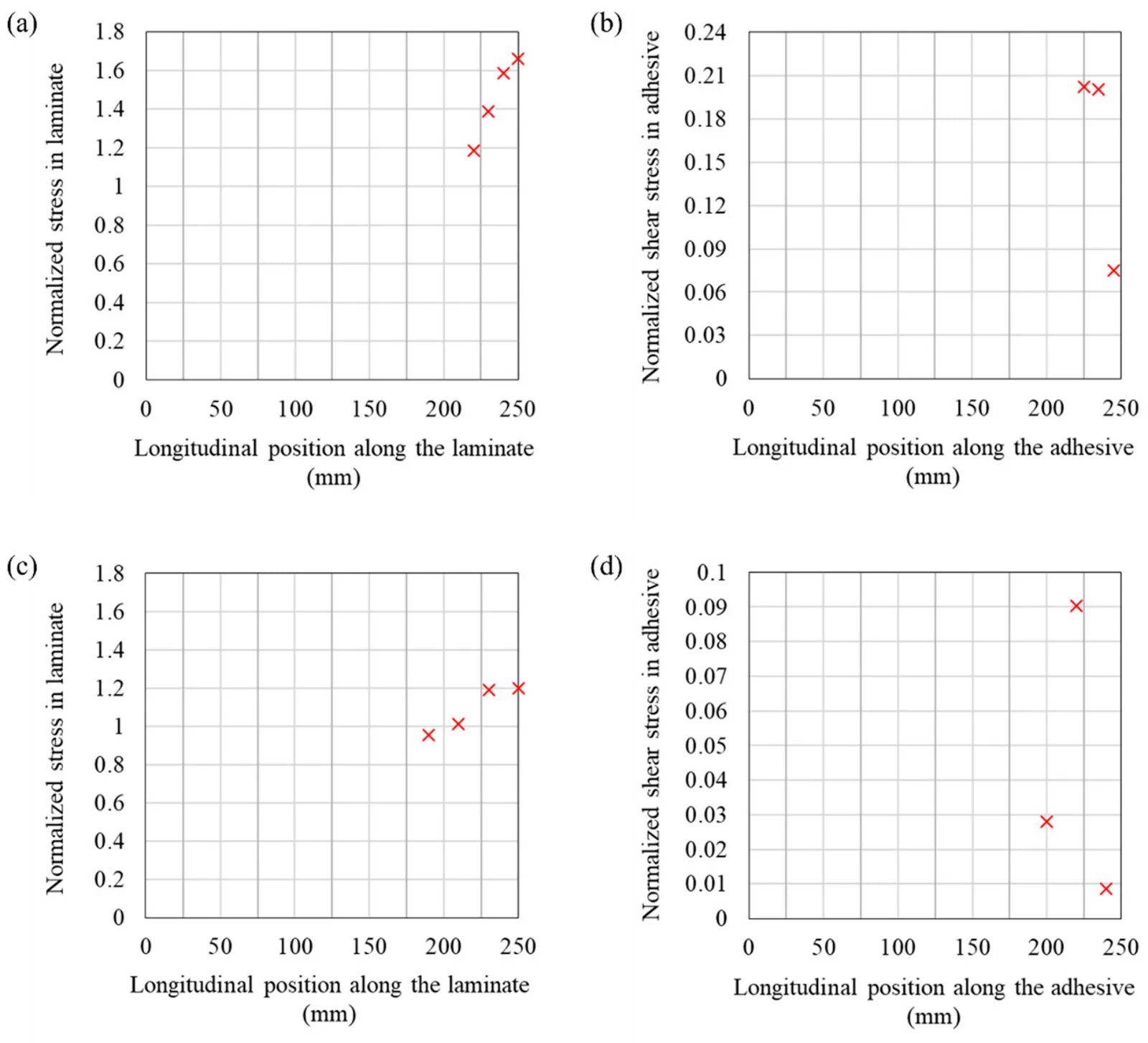
Normalized stress calculated based on strain gauge measurements. (**a**) Normalized normal stress in the bottom laminate and (**b**) normalized shear stress in the corresponding adhesive of a horizontally oriented beam. (**c**) Normalized normal stress in the outer laminate and (**d**) normalized shear stress in the corresponding adhesive of a vertically oriented beam.

Furthermore, the strain gauge measurements were used to determine the stress in the laminate and the adhesive stress in the glue according to Equations (5) and (6). It was then normalized with respect to the maximum normal stress at the bottom of the beam. The maximum normal stress was determined by Equations (19) and (20), assuming a homogeneous beam with a quadratic cross-section:

(19) $$M_{max}=\frac{P}{2}d$$

(20) $$\sigma_{max}=\frac{M_{max}\frac{t}{2}}{I}$$

where P is the axial load applied to the test rig, d denotes the distance between the point loads, I is the moment of inertia, and t is the thickness of the beam. Figure 18 summarizes the normalized stress in the laminate and the normalized shear stress in the corresponding adhesive for horizontally and vertically positioned beams, respectively.

### 3.2. Analytical Predictions

**Figure 19:**
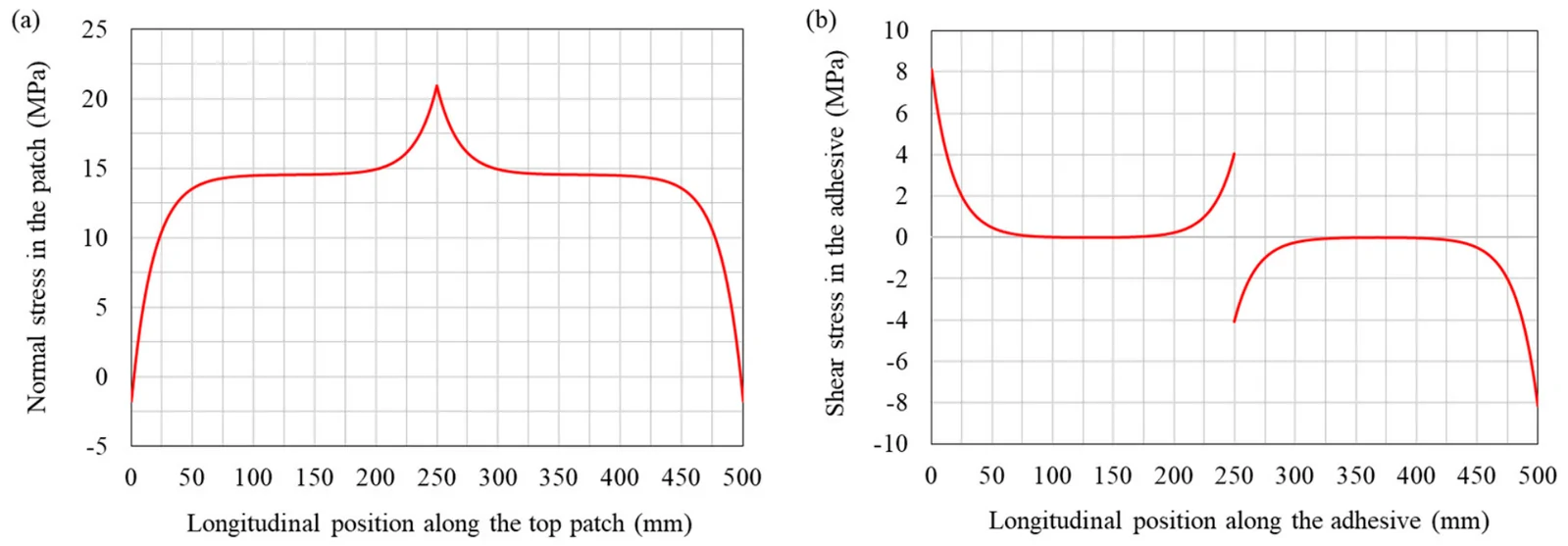
(**a**) Normal stress distribution in the patch and (**b**) shear stress distribution in the adhesive for the adhesive joint with 1 mm glue thickness when subjected to tension. The predictions were based on the direct linear-elastic solution for a double-doubler joint.

According to the modified analytical solutions for a double-doubler joint, stress distributions were predicted for the proposed adhesive joints subjected to tensile. Here, normal stress distribution in the patch and shear stress distribution in the adhesive for the joint with 1 mm glue are illustrated in Figure 19. The predictions for the adhesive joint with 4 mm glue are presented together with the experimental and numerical results in Section 4.1.

It is evident from both plots that stress distributions were symmetric about the geometric center due to the symmetry assumption in this one-dimensional analytical model. For normal stress distribution within the patch, stress was near zero at both ends where overlap started. Since no direct load transfer was theoretically expected at the butt-end joint, the applied load was instead transferred from the substrate to the patch through the adhesive layer and generated shear stress concentrations. This load-transfer mechanism induced a rapid stress increase in the patch near the overlap boundaries, culminating in a local maximum toward the geometric center of the adhesive joint. In correspondence, the adhesive shear stress experienced peaks at the overlap edges, including the central butt-end region. Between these peak values, shear stress declined toward a minimum, reflecting a more uniform and efficient load-transfer zone between the overlap boundaries and the butt-end joint.

**Figure 20:**
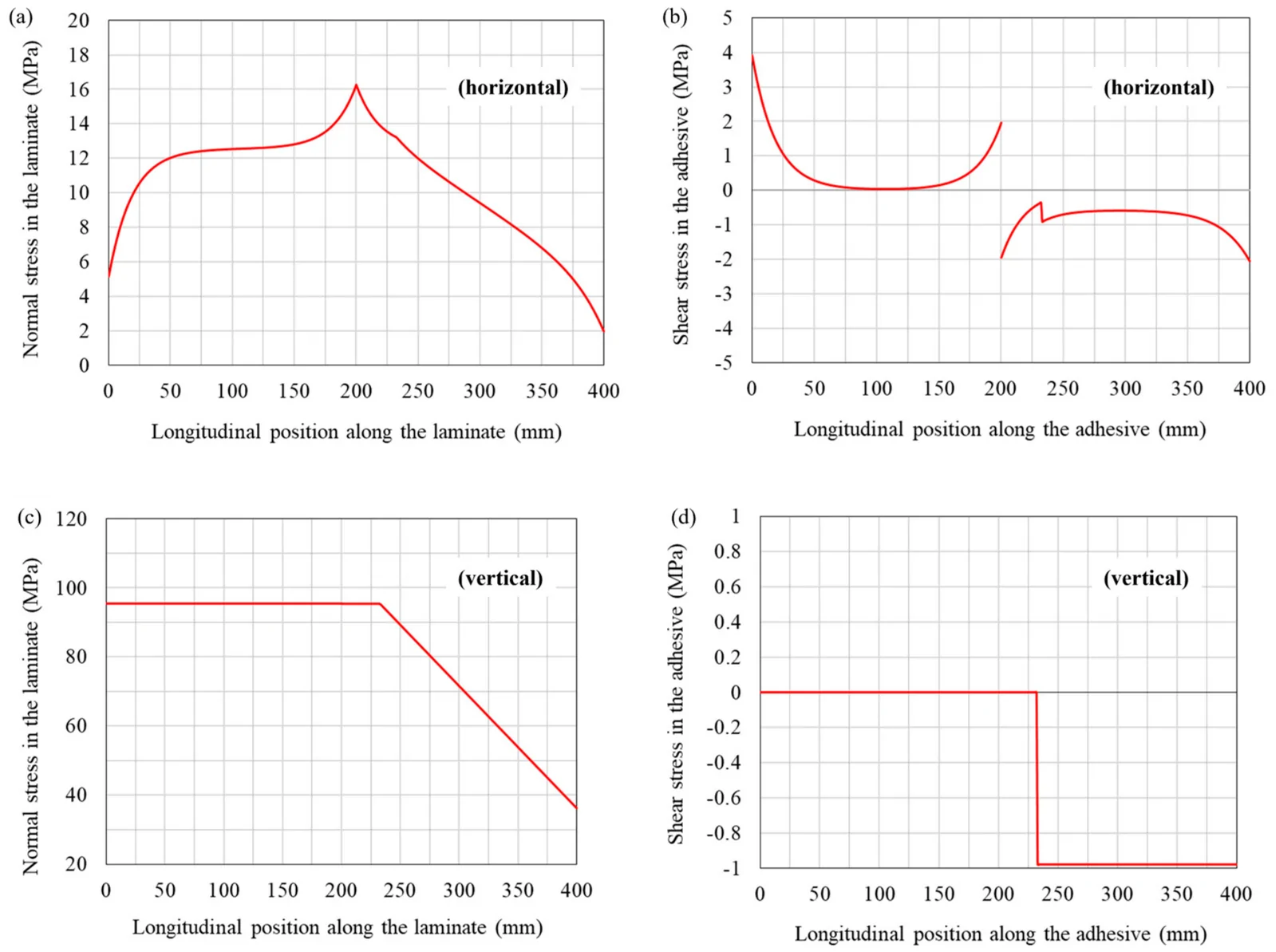
Based on the analytical framework, stress distributions were predicted for the beams under four-point bending. (**a**) Normal stress distribution in the bottom long laminate and (**b**) shear stress distribution in the corresponding adhesive layer of the horizontal beam. (**c**) Normal stress distribution at the bottom edge of the outer long laminate and (**d**) shear stress distribution in the corresponding adhesive layer of the vertical beam.

According to the analytical solutions developed for glued beams, stress distributions in laminate and adhesive are presented in Figure 20. Unlike the adhesive joint, stress distributions in the beam were not symmetrical due to the asymmetric loading conditions. It could be observed that stress distributions up to the loading point (x=232.5 mm) exhibited shapes similar to the adhesive joint. However, the stress in the laminate decreased at a relatively steady rate between the point load and the adjacent support (232.5 mm≤x≤400 mm). Additionally, adhesive shear stress experienced an abrupt increase at the loading location and maintained a higher magnitude in the region between the load and the support. These phenomena were attributed to the decreasing bending moment and the presence of a non-zero shear force in this segment of the beam. It is also noteworthy that, in the bottom laminate, stress did not initiate from zero at either end owing to the global bending moment contribution.

In the vertically beam configuration, stress distributions were governed primarily by global bending. Consequently, normal stress in the GFRP laminate and the adhesive shear stress followed the bending moment and shear force diagrams in Figure 10. Within constant bending moment zone (0≤x≤232.5 mm), the laminate experienced a uniform stress of approximately 95 MPa. At the location of the point load, the normal stress decreased linearly corresponding to the reduction in bending moment. The adhesive shear stress was zero within the zero-shear region (0≤x≤232.5 mm), then dropped abruptly to approximately −1 MPa at the point load and remained constant thereafter.

### 3.3. Finite Element Simulations

**Figure 21:**
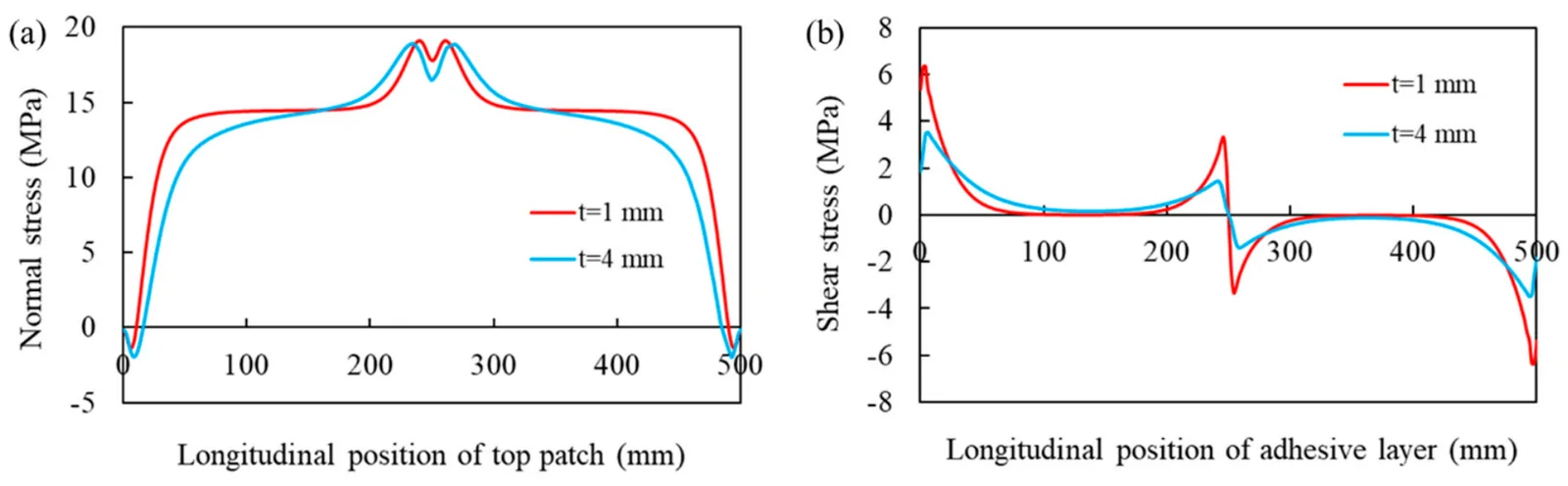
Stress distribution comparisons between adhesive joints with a glue thickness of 1 mm and 4mm: (**a**) normal stress distributions in the patch which was extracted from the middle of the outer surface of the top patch; (**b**) shear stress distributions in the adhesive, extracted from the middle of the adhesive surface that directly contact the top patch.

Normal stress distribution of the top patch was evaluated along the mid-line of its outer surface, while the corresponding shear stress distribution in the adhesive was obtained along the mid-line of the adhesive surface in contact with the top patch. Figure 21 compares the stress distributions between the adhesive joints with glue thicknesses of 1 mm and 4 mm. Figure 21a demonstrates that the glue thickness had negligible influence on normal stress distribution in the patch. For this symmetric double-doubler joint, normal stress distribution in the patch was primarily governed by the stiffness of GFRP and overall load transfer. As the adhesive was much more compliant than GFRP, slight variation in its thickness mainly influences local shear deformation without significantly altering the axial load path. Furthermore, a sudden drop in the mid-region was observed for both adhesive joints. This could be attributed to shear lag effect as well as the eccentric force discussed in Section 2.3.1.

Though the shear stress distributions (see Figure 21b) yielded similar profile, the glue experienced higher peak shear stress in the adhesive joint with thinner glue. This is caused by a lower shear stiffness of a thicker adhesive layer, allowing greater deformation in the glue and more uniform load transfer along the bonded interface.

**Figure 22:**
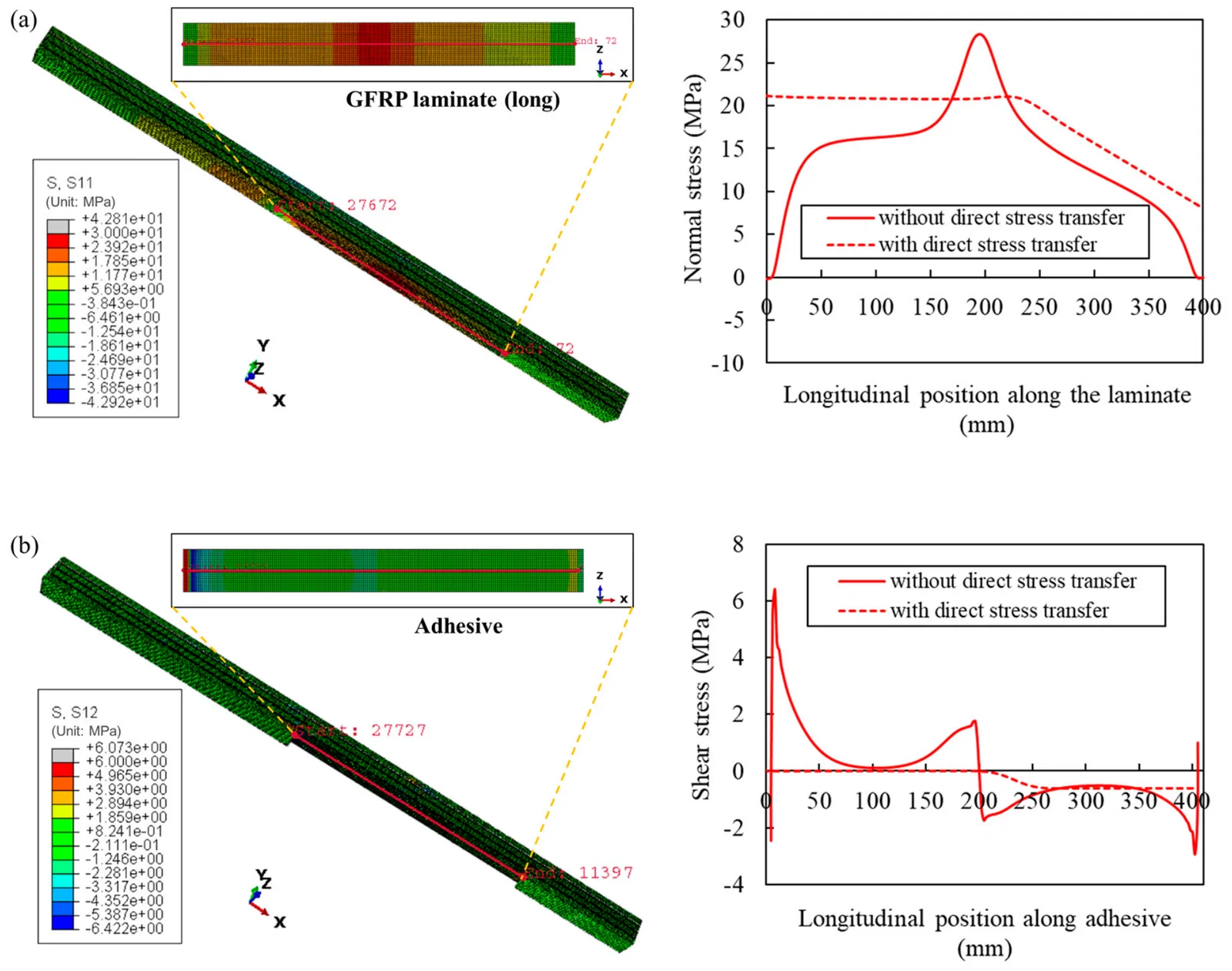
Results from FE models of the horizontally positioned beam subjected to bending: (**a**) normal stress distribution at the bottom of the long laminate, extracted from FE model and plotted in the graph on the right; (**b**) shear stress distribution in the adhesive that contacted the long laminate, extracted from FE model and plotted in the graph on the right. It should be noted that the color-contoured stress distributions correspond to the case without direct stress transfer between laminates within the same layer.

For the horizontally placed beam, normal stress was obtained from the FE models along the mid-line of the bottom laminate (see Figure 22a) where strain gauges were located. Correspondingly, the shear stress distribution in the adhesive layer directly above this laminate was examined along the longitudinal mid-line (see Figure 22b).

For the scenario without direct stress transfer between laminates within the same layer, the bottom long laminate examined here played a similar role as the patch in an adhesive joint. Hence, the normal stress distribution shown in Figure 22a resembled the stress distribution of the patch element (see Figure 21a). However, no drop was observed in the normal stress distribution at the midsection. This is mainly due to the constraints from the upper joints and adjacent laminates in a multi-joint glued beam, which reduced the localized stress variations associated with shear lag effect. While the laminates carried the axial load, the adhesive layers transferred the load from one laminate to the other via shear deformation. As illustrated in Figure 22b, shear stress in the adhesive layer peaked near the overlap ends where load transfer initiated and terminated. Progressing toward the overlap center, the load difference between laminates decreased due to stress redistribution. This resulted in a smooth decline in shear stress.

For the scenario with direct stress transfer between laminates within the same layer, normal stress distribution along the bottom laminate followed the bending moment diagram (see Figure 10). To be specific, stress was uniform within the constant-moment region (0≤x≤232.5 mm) and decreased linearly from the loading point toward the right support (232.5≤x≤400 mm). Similarly, adhesive shear stress distribution resembled shear force diagram (see Figure 10). In the zero-shear region (0≤x≤232.5 mm), shear stress was negligible. Then, it increased gradually from the loading point and remained approximately constant, reflecting the constant shear force in this region.

**Figure 23:**
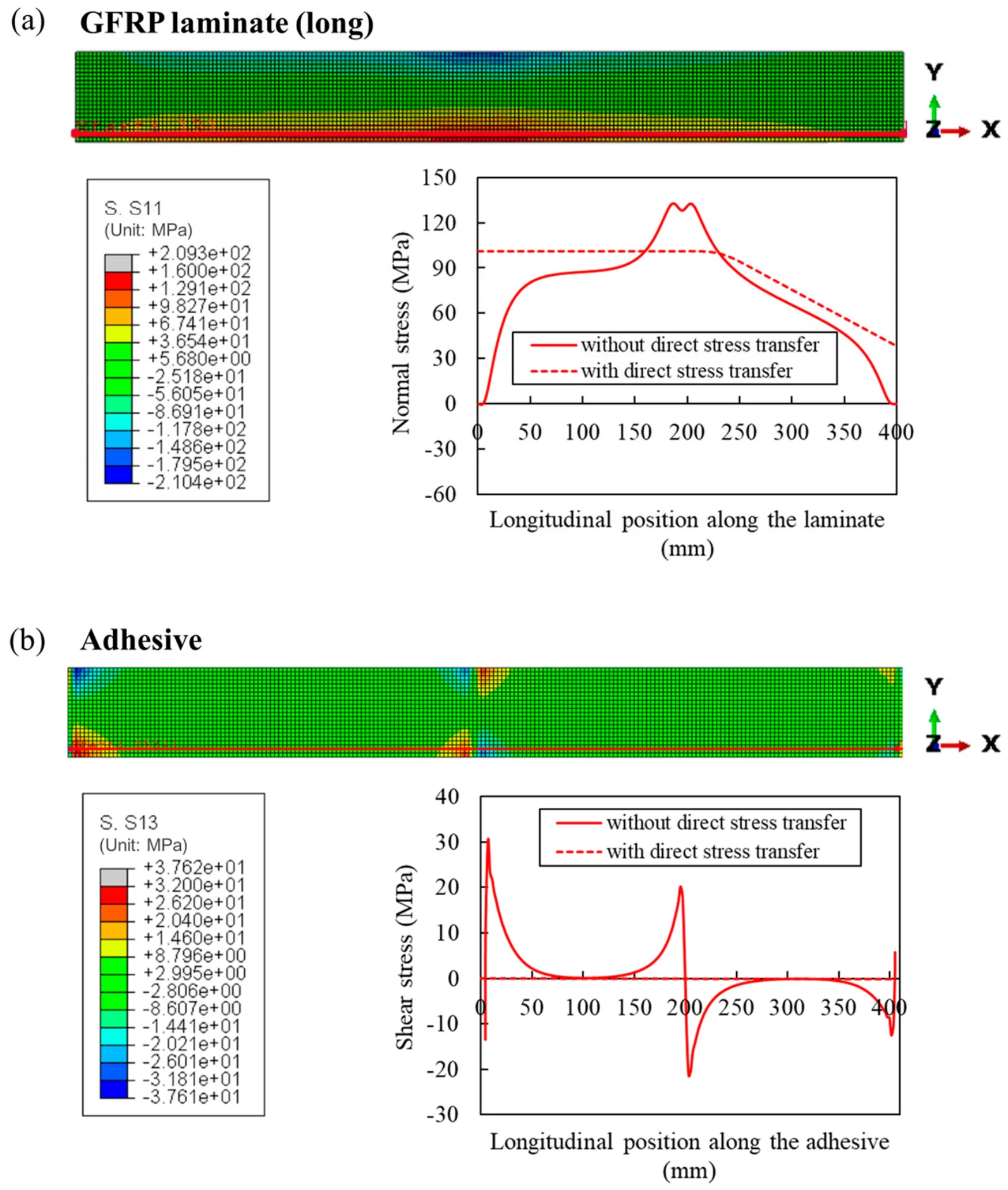
Results from FE model of the vertically positioned beam subjected to bending: (**a**) normal stress distribution close to the bottom of the outer long laminate, extracted from FE model and plotted in the graph; (**b**) shear stress distribution in the adhesive that contacted the long laminate, extracted from FE model and plotted in the graph on the right. It should be noted that the color-contoured stress distributions correspond to the case without direct stress transfer between laminates within the same layer.

For the vertically oriented beam, the normal stress distribution of the outer long laminate, as well as the shear stress distribution of the corresponding adhesive, were extracted the same way as described for the horizontally positioned beam. Results are presented in Figure 23.

## 4. Discussions

To investigate the stress distributions in the adhesive joints and GL-FRP beams, results from experiments, analytical solutions, and FE simulations are compared. The capability of FE models and analytical frameworks in terms of stress predictions are also evaluated.

### 4.1. Stress Distribution in Adhesive Joints

**Figure 24:**
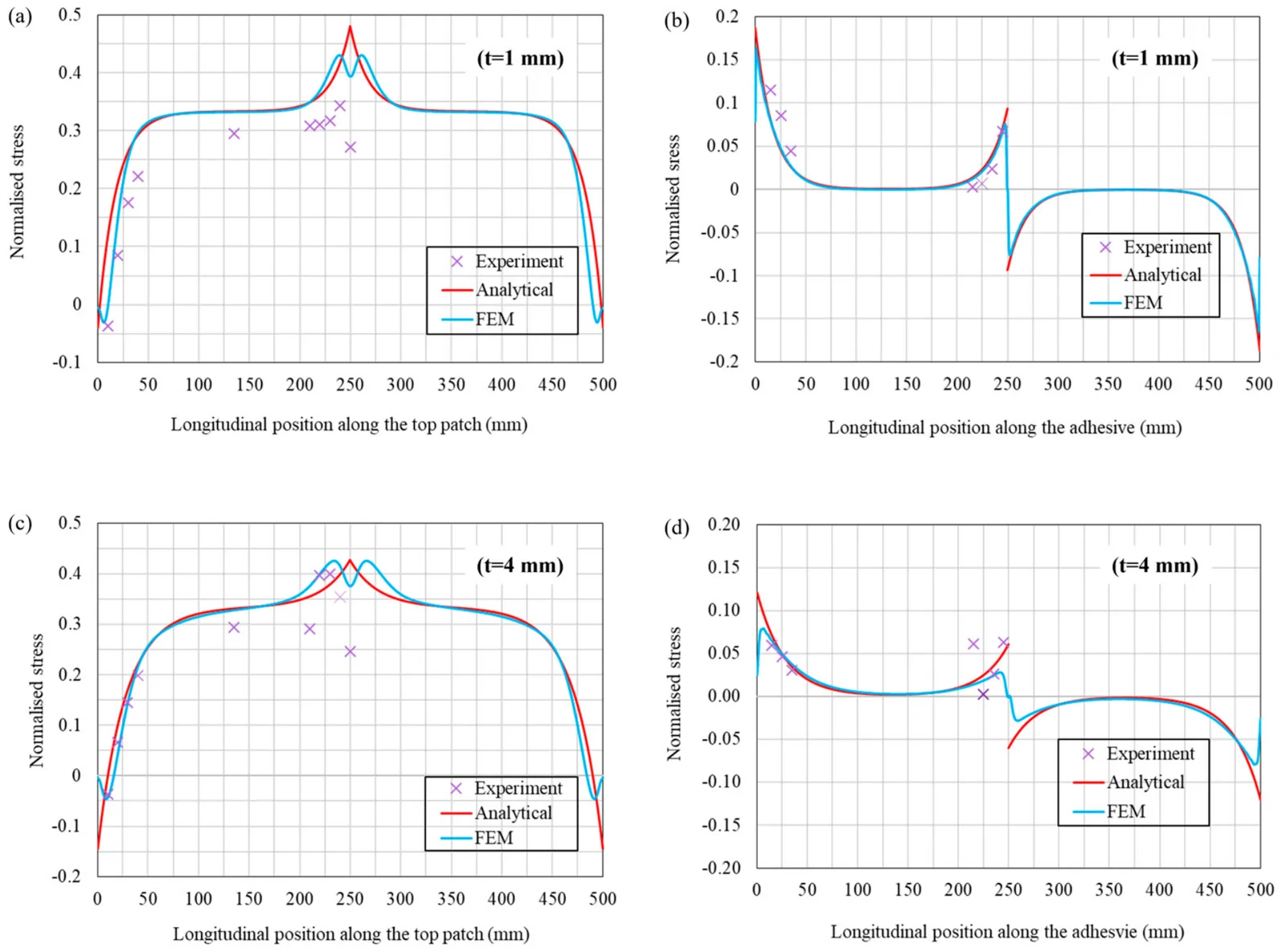
Comparison among experimental results, analytical solutions, and FE simulations of adhesive joint tests. (**a**) Normalized stress distribution in the patch and (**b**) normalized shear stress distribution in the adhesive for adhesive joint with glue thickness of 1 mm. (**c**) Normalized stress distribution in the patch and (**d**) normalized shear stress distribution in the adhesive for adhesive joint with glue thickness of 4 mm.

Figure 24 presents a comparison of the experimental, analytical, and FE results of adhesive joint tests. To ensure the results were comparable, stress was normalized for all cases. Overall, the stress distribution profiles predicted by FE models, analytical frameworks matched the stress obtained from experiment. For the patch element, the axial stress was initially zero at the overlap edges. Theoretically, there was no load transfer at the butt-end joint. Thus, load was transferred from the substrate elements to the patch through the adhesive, generating concentrated shear stress in the adhesive. This transition led to a rapid increase in normal stress within the patch, resulting in a sharp rise near the edges and a local maximum near the center. Correspondingly, the normalized shear stress reached its local maximums at the overlap edges (including the middle butt-end joint). Between the peaks, the adhesive shear stress decreased and approached its minimum. This indicated a more uniform load transfer between the overlap edges and the butt-end joint.

It can be seen from Figure 24a,c that the normalized stresses measured in the patch were lower than those predicted by analytical model and FE simulation. The discrepancy is likely caused by slight pre-bending of the specimens. During experiment, the straightening of the adhesive joints could alter the initial strain readings and affect other gauge measurements. Additionally, deviations may also arise from stress transfer between substrate elements across the butt-end joint. Even though Teflon was used between the substrate elements, the possibility of load transfer still existed. For normalized shear stress, the experimental results agreed well with the models near the ends but deviated in the midsection, as illustrated in Figure 24b,d. As shear stress was determined by the difference in normal stress between adjacent strain gauges, an accurate estimation of shear stress required minimal spacing between the gauges. Consequently, shear stress obtained from experiments could deviate from values estimated using direct linear-elastic model and FE simulation.

The influence of adhesive layer thickness was evident from strain gauge measurements, analytical predictions, and FE models. A thicker adhesive layer resulted in a lower joint stiffness, allowing greater shear deformation within the adhesive. This increased compliance led to a longer and more gradual load transfer between the patch and substrate elements. It consequently reduced the rate at which shear stress and normal stress developed near the overlap ends. Hence, the adhesive joint with thicker glue yielded less steep shear stress gradient and lower peaks (see Figure 24b,d). A similar phenomenon could be observed for stress distribution in the patch, although the effect is less pronounced due to its higher stiffness than adhesive.

Furthermore, the normalized stress in the patch extracted from FE models (Figure 24a,c) and obtained from the experiment both experienced a sudden drop in the middle. This could be from the shear lag effect. In other words, the normal stress in the patch increased rapidly when the load was transferred from the adhesive, reached peak value, and then decreased once the load was fully distributed. Another reason could be the eccentric force discussion in Section 2.3.1. As the force in the patch was transferred through shear in the adhesive joint, it could be considered as an eccentric force acting on the outer surface of the patch. The eccentricity then resulted in a moment inducing compression in the outer surface of the patch. As a result, the stress in the patch experienced a local decrease from both FE simulations and strain gauge measurements. Analytical models accounted for both effects but to a lesser extent than the FE models.

### 4.2. Stress Distribution in GL-FRP Beams

**Figure 25:**
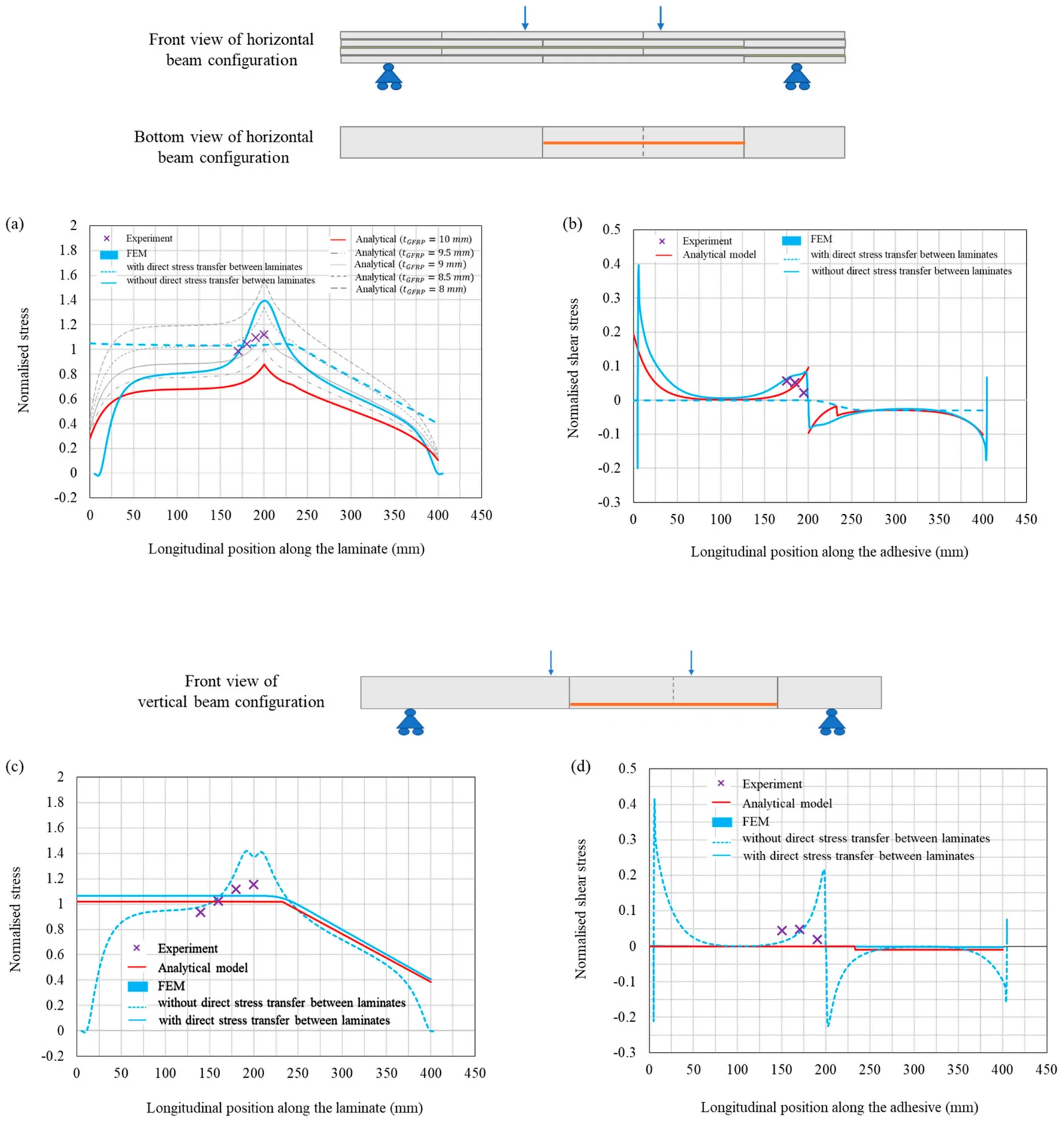
Comparisons among experimental results, analytical solutions, and FE simulations of four-point bending tests. Beam configurations were schematically presented corresponding to the stress distributions. The orange lines in the beam drawings represent the locations where the stress distributions were examined. (**a**) Normalized stress distribution in the bottom long laminate and (**b**) normalized shear stress distribution in the adhesive for beam positioned horizontally. (**c**) Normalized stress distribution in the outer laminate near the bottom edge and (**d**) normalized shear stress distribution in the adhesive for beam positioned vertically.

To study the flexural behavior of glued beams, Figure 25 compares experimental, analytical, and FE results from the bending tests. Normalized stress distribution in laminate and normalized shear stress distribution in the adhesive are summarized for both horizontal and vertical beam configurations.

In general, FE results showed good agreement with experimental measurements regardless of beam orientations. As shown in Figure 25, stress derived from strain gauge data mostly fell within the ranges predicted by the numerical simulations. The FE analysis provided stress distributions for two extreme scenarios of interfacial behavior between the laminates within the same layer. In practice, imperfections at the butt-end joints allowed partial stress transfer between laminates so that the experimental measurements lay within the ranges approximated by the numerical predictions. The only exception was the adhesive shear stress in the vertical beam configuration (see Figure 25d), where one normalized experimental value slightly exceeded the FE-predicted range. As shear stress was calculated based on the difference in normal stress between adjacent gauges, its accuracy largely depended on small gauge spacing. Thus, shear stress obtained from tests could deviate from FE predictions. However, given the low magnitude of shear stress level, this discrepancy is negligible.

For the horizontal beam, the proposed analytical model underestimated normal stress in the laminate compared to experimental and numerical results (see Figure 25a). On the other hand, the analytically predicted shear stress corresponded well to both experiments and FE results (see Figure 25b). The laminate normal stress was very sensitive to local effects such as load eccentricity, peel effects, and assembly imperfections. These localized effects were not fully represented in the one-dimensional linear-elastic model. In contrast, adhesive shear stress was governed mainly by the average in-plane load transfer through the glue and was less affected by local effects. For instance, peel stress was not considered in the proposed analytical solutions. However, it could induce local bending and out-of-plane deformation, which increased the stress in the laminate. On the other hand, peel stress had only a local influence near the joint ends and did not largely affect the global shear stress distribution. Therefore, the analytical model predicted adhesive shear stress reasonably accurate though it underestimated laminate normal stress.

To assess the feasibility of the analytical model developed for normal stress in the laminate, predicted normal stress distributions for horizontal beams with different laminate thicknesses are also presented in Figure 25a. Significant variations in normal stress were observed with minor changes in laminate thickness. The analytical prediction for a 9 mm laminate thickness showed excellent agreement with experimental measurements and FE predictions. This could be attributed to manufacturing tolerances of GFRP laminates from the factory, which results in thinner laminate than the nominal 10 mm thickness. Furthermore, the simplified analytical model does not account for peel and out-of-plane shear. The thickness parameter acts as an ‘effective thickness’ rather than purely geometric parameter. While the modified direct linear-elastic model could capture the overall shape of normal stress distributions, the analytical predictions exhibited high sensitivity to laminate thickness. This sensitivity highlights a limitation in the simplified analytical model. Therefore, the analytical predictions require calibration accordingly, and the calculations in practice should be approached with caution.

For the vertical beam configuration, the analytical predictions of normal stress distribution in the laminate closely matched the FE simulation scenario assuming direct stress transfer between laminates (see Figure 25c). The analytically predicted stress mostly lay within the bounds defined by the two FE scenarios, except in the middle section. Comparison with the strain gauge measurements also revealed discrepancies: the experimental results indicated an increasing stress toward the middle, whereas the analytical predictions remained constant. The deviations could arise from the assumption in the analytical model that vertical beam was subjected to pure bending. This assumption implied perfect bonding and uniform, fully effective load transfer through the adhesive. In contrast, the FE models captured stress redistribution near the butt-end joint, where partial interaction developed between the adjacent laminates within the same layer. This behavior was not accounted for in analytical formulation, leading to discrepancies from experiments and numerical simulations. The results indicate that the response of vertical beam was governed by a combination of bending, interlaminar shear, and partial interaction between the laminates. Therefore, failure of the vertical beam is expected to involve multiple mechanisms, allowing progressive stress redistribution prior to failure and preventing sudden catastrophic rupture in brittle FRP laminates.

Furthermore, Figure 25d compares adhesive shear stress distributions in the vertical beam. Consistent with the normal stress distribution, the analytical predictions agreed well with the FE scenario with direct stress transfer between laminates. The normalized shear stress remained close to zero, indicating a bending-dominated response. This phenomenon is further supported by the tensile rapture failure recorded in the vertical beam (see Figure 16b).

The above comparisons reveal that horizontal beam (see Figure 25a,b) and vertical beam (see Figure 25c,d) showed distinct structural responses under four-point bending. While the horizontal beam was primarily governed by interlaminar shear stress transfer through the adhesive layers, the vertical beam was dominated by global bending. As a result, the horizontal beam is expected more sensitive to bond quality, whereas the vertical beam is more dependent on consistent laminate properties. This difference plays an important role in beam design and fabrication processes to optimize its strength and reliability.

## 5. Conclusions

In this study, a novel concept for upcycling decommissioned wind turbine blades into GL-FRP beams was presented and assessed. The concept was based on extracting shell segments from the turbine blades and bonding them into load-bearing beam configurations, providing a viable and scalable pathway for high-value reuse in the construction sector.

The preliminary study conducted here aimed to evaluate the feasibility of this approach. It was carried out at both the adhesive joint and beam levels, examining local joint behavior under tensile loading and global flexural performance of glued beams under bending. Based on these investigations, the following conclusions can be drawn:

- Mechanical roughening of the laminate surfaces largely improved interfacial adhesion and the load-bearing capacity of the adhesive joints. However, the differences in surface roughness produced by P80 and P240 sandpapers had a negligible effect.
- For adhesive joints, normal stress distribution in the patch were well captured by FE simulations and the direct linear-elastic model was validated by experiments. The stress was initially zero at the overlap edges, then increased rapidly to a local maximum near the center. Local effects, such as shear lag and eccentric loading, were demonstrated to be more effectively represented in FE models than analytical solution.
- For adhesive joints, adhesive shear stress peaked at the overlap edges (including the butt-end joint), while decreasing between these locations.
- The GL-FRP beams yielded higher load-bearing capacity in vertical configuration (~20 kN) than horizontal configuration (~8 kN) under four-point bending. This was attributed to the smaller moment of inertia of the critical cross-section in horizontal beam.
- The structural response of horizontal GL-FRP beam was primarily governed by interlaminar shear transfer, making it highly sensitive to bond quality. On the other hand, global bending governed the vertical beam, with its strength and reliability largely dependent on uniform laminate properties.
- FE models for GL-FRP beams, without direct stress transfer, accurately captured the overall shape of stress distributions regardless of beam orientations. Due to imperfections at the butt-end joints in beam specimens, experimental measurements reasonably fell within the bounds defined by the two extreme FE scenarios.
- The proposed analytical framework for GL-FRP beams reliably predicted adhesive shear stress for both horizontal and vertical configurations. In terms of stress in the laminate, the analytical solution requires further calibration for horizontal beam. Besides, the pure bending assumption for vertical beam resulted in some discrepancy in analytical predictions of laminate stress.

While the structural feasibility of the proposed GL-FRP beams was demonstrated, the use of commercial GFRP laminates presents certain limitations. Actual turbine blades feature complex biaxial/triaxial GFRP and balsa/PVC foam core materials, alongside in-service fatigue, moisture, and UV degradation. Hence, investigating the GL-FRP beam, which is made from real decommissioned wind turbine blades, is a critical next step required to safely translate this upcycling framework into practical civil engineering applications. Furthermore, future research should also assess the impacts of long-term mechanical fatigue, moisture degradation, and environmental aging on these reclaimed materials to ensure their reliability over an extended service life.

## Figures and Tables

**Table 2:** Material properties of GFRP laminate according to the datasheet provided by Fiberline [50].

Material Property	Characteristic Value
Tensile modulus [GPa]	24.0
Shear modulus [GPa]	3.0
Poisson’s ratio [-]	0.23
Tensile strength [MPa]	240.0
Compressive strength, axial [MPa]	240.0
Flexural strength, axial [MPa]	240.0
Interlaminar shear strength [MPa]	20.0

**Table 3:** Material properties of the adhesive according to the datasheet provided by ITW Performance Polymers [51].

Material Property	Value *
Tensile modulus [MPa]	1034–1207
Shear modulus ** [MPa]	800–875
Tensile strength [MPa]	24.0–31.0
Cohesive (shear) strength *** [MPa]	20.7–24.1

Note: * ‘Value’ refers to as “Typical Mechanical Properties” in datasheet. ** Provided through contact to the retailer HF Industri & Marine ApS who were in contact with ITW Performance Polymers *** For a 0.30 mm gap.

**Table 4:** Material properties of GFRP laminates measured from six tensile tests.

Material Property	Mean Value	Standard Deviation	Alpha	COV	Characteristic Value
Tensile modulus [GPa]	32.16	0.95	0.05	0.03	32.12
Tensile strength [MPa]	342.99	21.79	0.05	0.06	298.5
Poisson’s ratio [-]	0.32	0.04	0.05	0.12	0.32

**Table 5:** Four designs of adhesive joints with different surface treatment and glue thickness.

Design	Surface Roughness *	Adhesive Thickness $t_{a}$(mm)	Number of Specimens
1	Untreated	1	3
2	P80	1	3
3	P240	1	3
4	P80	4	2

* Note: surface roughness in the table is indicated by the grade of sandpaper that was used to treat the surface of the laminates.

## Data Availability

The original contributions presented in this study are included in the article. Further inquiries can be directed to the corresponding author.
